# A protein evolution model with independent sites that reproduces site-specific amino acid distributions from the Protein Data Bank

**DOI:** 10.1186/1471-2148-6-43

**Published:** 2006-05-31

**Authors:** Ugo Bastolla, Markus Porto, H Eduardo Roman, Michele Vendruscolo

**Affiliations:** 1 Centro de Biología Molecular "Severo Ochoa", (CSIC-UAM), Cantoblanco, 28049 Madrid, Spain; 2 Institut für Festkörperphysik, Technische Universität Darmstadt, Hochschulstr. 8, 64289 Darmstadt, Germany; 3 Dipartimento di Fisica, Università di Milano Bicocca, Piazza della Scienza 3, 20126 Milano, Italy; 4 Department of Chemistry, University of Cambridge, Lensfield Road, Cambridge CB2 1EW, UK

## Abstract

**Background:**

Since thermodynamic stability is a global property of proteins that has to be conserved during evolution, the selective pressure at a given site of a protein sequence depends on the amino acids present at other sites. However, models of molecular evolution that aim at reconstructing the evolutionary history of macromolecules become computationally intractable if such correlations between sites are explicitly taken into account.

**Results:**

We introduce an evolutionary model with sites evolving independently under a global constraint on the conservation of structural stability. This model consists of a selection process, which depends on two hydrophobicity parameters that can be computed from protein sequences without any fit, and a mutation process for which we consider various models. It reproduces quantitatively the results of Structurally Constrained Neutral (SCN) simulations of protein evolution in which the stability of the native state is explicitly computed and conserved. We then compare the predicted site-specific amino acid distributions with those sampled from the Protein Data Bank (PDB). The parameters of the mutation model, whose number varies between zero and five, are fitted from the data. The mean correlation coefficient between predicted and observed site-specific amino acid distributions is larger than <*r*> = 0.70 for a mutation model with no free parameters and no genetic code. In contrast, considering only the mutation process with no selection yields a mean correlation coefficient of <*r*> = 0.56 with three fitted parameters. The mutation model that best fits the data takes into account increased mutation rate at CpG dinucleotides, yielding <*r*> = 0.90 with five parameters.

**Conclusion:**

The effective selection process that we propose reproduces well amino acid distributions as observed in the protein sequences in the PDB. Its simplicity makes it very promising for likelihood calculations in phylogenetic studies. Interestingly, in this approach the mutation process influences the effective selection process, i.e. selection and mutation must be entangled in order to obtain effectively independent sites. This interdependence between mutation and selection reflects the deep influence that mutation has on the evolutionary process: The bias in the mutation influences the thermodynamic properties of the evolving proteins, in agreement with comparative studies of bacterial proteomes, and it also influences the rate of accepted mutations.

## Background

The evolutionary information embedded in the sequences of extant biological macromolecules can be used to reconstruct their evolutionary history (see for instance Ref. [1,2]). Methods based on the Maximum Likelihood (ML) principle are quite successful in reconstructing the past of molecules and species [3], but they rely on models of the evolutionary process at the molecular level. ML computations are usually carried out assuming that the sites of a protein evolve independently, a feature that is rather unrealistic, since selection for thermodynamic stability of the native state acts on all sites at the same time, introducing correlations between sites [4,5].

Incorporating selection for thermodynamic stability in a ML framework has been recently the focus of very interesting studies [6-10]. However, for large data sets ML computations become unfeasible without the assumption of independent sites. It has been also shown that modeling site-specific residue frequencies significantly improves methods for evolutionary reconstructions [11].

The simplest models of molecular evolution are based only on the mutation process and do not attempt to evaluate its effect on fitness. Kimura's neutral model [12,13] uses a binary fitness function to represent purifying selection. Protein sequences are considered either unviable or equivalent (neutral). In this model a fraction *x *of the mutations gives rise to neutral mutants and the remaining fraction 1 - *x *is eliminated by purifying selection.

In this paper, we use a binary fitness function based on the evaluation of the thermodynamic stability of the native state, in the same spirit of models first introduced in the context of RNA evolution [14-16] and subsequently extended to protein evolution [6,17-28]. The model that we study is a neutral evolution model with explicit stability requirements, the Structurally Constrained Neutral (SCN) model of protein evolution [29-32]. We study the model in the limit in which the product of the population size *M *times the mutation rate *μ *is small, which means that the population is very narrowly distributed in genotype space. This limit is appropriate for animal populations. In the regime of frequent mutation, the evolutionary dynamics is different from the rare mutation regime considered here in that there is a trend towards increased mutational robustness for increasing *Mμ *[33-35].

Stability requirements induce correlations between the sites of the macromolecule, as the free energy is a property of the system as a whole. In the present paper, we present an evolutionary model in which the sites evolve independently of each other and, in addition, are subject to structural stability. We show that the selection rules can be chosen in such a way to reproduce the evolutionary process simulated through the SCN model of protein evolution, in which the stability is explicitly evaluated. In order to eliminate the correlations induced by stability requirements there is, however, a price to pay: In the mean-field model, the effective selection depends on the mutation process. As a result, sites evolve independently but selection and mutation become interrelated, whereas in real evolution sites evolve in a correlated fashion and mutation and selection are independent processes.

We simulated the SCN model with different mutation schemes. The results of these simulations agree very well with the mean-field model, and show that both mutation and selection have influence on protein folding thermodynamics.

Furthermore, we applied the effective evolutionary model to a non-redundant set of globular protein structures contained in the Protein Data Bank (PDB), using several mutation schemes of increasing complexity. The mutation scheme that best reproduces the observed amino acid distributions at all sites is one that takes into account the increase of the mutation rate at CpG dinucleotides. The site-specific amino acid distributions obtained through this model reproduce quite well observed amino acid distributions.

### The SCN model

The SCN model evaluates the "fitness" associated with a protein sequence through a model of protein folding based on an effective free energy function (see Eq. (13) in Methods). We adopt two measures of protein folding stability: (1) with respect to the unfolded state (unfolding stability), estimated through the effective native energy, and (2) with respect to misfolded states (misfolding stabililty), estimated through the normalized energy gap (see Methods). We use a binary fitness function that assigns fitness one if both stabilities are above predefined thresholds and zero otherwise. Thus our model is neutral, since there is no fitness difference between viable proteins.

The free energy calculations introduce correlations between the sites of the protein. As a result of these dependencies, the fraction of neutral mutations *x *is no longer constant, as in Kimura's model, but fluctuates broadly from one sequence to another. This implies that the distribution of the number of substitutions is broader than the Poissonian distribution arising from the standard neutral model by Kimura, i.e. the process of protein evolution is overdispersed [30-32], in better agreement with empirical observations [36,37].

## Results

### Optimal sequence for a protein structure

We evaluate the stability of folded states through the contact free energy function

E(C,A)=∑ijCijU(Ai,Aj),     (1)
 MathType@MTEF@5@5@+=feaafiart1ev1aaatCvAUfKttLearuWrP9MDH5MBPbIqV92AaeXatLxBI9gBaebbnrfifHhDYfgasaacH8akY=wiFfYdH8Gipec8Eeeu0xXdbba9frFj0=OqFfea0dXdd9vqai=hGuQ8kuc9pgc9s8qqaq=dirpe0xb9q8qiLsFr0=vr0=vr0dc8meaabaqaciaacaGaaeqabaqabeGadaaakeaacqWGfbqrcqGGOaakieqacqWFdbWqcqGGSaalcqWFbbqqcqGGPaqkcqGH9aqpdaaeqbqaaiabdoeadnaaBaaaleaacqWGPbqAcqWGQbGAaeqaaOGaemyvauLaeiikaGIaemyqae0aaSbaaSqaaiabdMgaPbqabaGccqGGSaalcqWGbbqqdaWgaaWcbaGaemOAaOgabeaakiabcMcaPiabcYcaSiaaxMaacaWLjaGaeiikaGIaeGymaeJaeiykaKcaleaacqWGPbqAcqWGQbGAaeqaniabggHiLdaaaa@4A10@

where **C **represents the binary contact matrix derived from the protein structure, **A **represents the protein sequence and *U*(*a, b*) is the effective contact interaction strength between amino acids *a *and *b*, belonging to the set of twenty standard amino acids. This model of protein stability, despite its simplicity, captures several relevant features of protein folding, in particular those related with hydrophobicity. In particular, it allows to estimate the stability against unfolding and misfolding for sequence-structure pairs, in such a way that the native structure is more stable than all alternative structures for almost all protein chains in the PDB [38]. Difference in stability between homologous proteins can be related to evolutionary and ecological variables [39], and estimates of unfolding free energy are correlated with experimental measures (UB, unpublished result).

A further approximation of this model allows to design analytically the optimally stable sequence for a given fold. This approximation consists in truncating the spectral decomposition of the contact interaction matrix at the first spectral component, namely *U*(*a, b*) ≈ -*h*(*a*)*h*(*b*), where *h*(*a*) indicates the component of the main eigenvector of *U*(*a, b*) corresponding to amino acid *a*, which we call the *interactivity *of amino acid *a*. It is well known that the main eigenvector of contact interaction matrices is related to hydrophobicity [40,41]. Consistently, the interactivity scale is strongly correlated with empirical hydropathy scales as for instance the octanol scale derived by Fauchere and Pliska [42].

We define the hydrophobicity profile (HP) of a protein sequence associating at each site the interactivity value *h*(*A*_*i*_) corresponding to its amino acid. Using only the first spectral component, the interaction matrix can be reconstructed to good accuracy (the correlation coefficient between *U*(*a, b*) and -*h*(*a*)*h*(*b*) is *r *= 0.80). Under this approximation, the energy can be written as a quadratic form of the hydrophobicity profile

E(C,A)≈−∑ijCijh(Ai)h(Aj).     (2)
 MathType@MTEF@5@5@+=feaafiart1ev1aaatCvAUfKttLearuWrP9MDH5MBPbIqV92AaeXatLxBI9gBaebbnrfifHhDYfgasaacH8akY=wiFfYdH8Gipec8Eeeu0xXdbba9frFj0=OqFfea0dXdd9vqai=hGuQ8kuc9pgc9s8qqaq=dirpe0xb9q8qiLsFr0=vr0=vr0dc8meaabaqaciaacaGaaeqabaqabeGadaaakeaacqWGfbqrcqGGOaakieqacqWFdbWqcqWFSaalcqWFbbqqcqWFPaqkcqGHijYUcqGHsisldaaeqbqaaiabdoeadnaaBaaaleaacqWGPbqAcqWGQbGAaeqaaOGaemiAaGgaleaaieWacqGFPbqAcqGFQbGAaeqaniabggHiLdGccqGGOaakcqWGbbqqdaWgaaWcbaGaemyAaKgabeaakiabcMcaPiabdIgaOjabcIcaOiabdgeabnaaBaaaleaacqWGQbGAaeqaaOGaeiykaKIaeiOla4IaaCzcaiaaxMaacqGGOaakcqaIYaGmcqGGPaqkaaa@4E05@

In the following, we will use the complete contact interaction matrix *U*(*a, b*), Eq. (1), for simulations of the SCN model, and the hydrophobic energy Eq. (2) for analytic computations. We further neglect the discretization of the HP into twenty values and consider the *h_i _*as real valued independent variables. This setting allows to solve analytically the sequence design problem of determining the optimally stable sequence for a target structure. The HP with minimal energy for a given contact matrix **C **and for fixed mean square^1 ^∑ihi2/N≡〈h2〉
 MathType@MTEF@5@5@+=feaafiart1ev1aaatCvAUfKttLearuWrP9MDH5MBPbIqV92AaeXatLxBI9gBaebbnrfifHhDYfgasaacH8akY=wiFfYdH8Gipec8Eeeu0xXdbba9frFj0=OqFfea0dXdd9vqai=hGuQ8kuc9pgc9s8qqaq=dirpe0xb9q8qiLsFr0=vr0=vr0dc8meaabaqaciaacaGaaeqabaqabeGadaaakeaadaaeqaqaaiabdIgaOnaaDaaaleaacqWGPbqAaeaacqaIYaGmaaGccqGGVaWlcqWGobGtcqGHHjIUcqGHPms4cqWGObaAdaahaaWcbeqaaiabikdaYaaakiabgQYiXdWcbaGaemyAaKgabeqdcqGHris5aaaa@3DA0@ is parallel to the principal eigenvector (PE) of the contact matrix, whose components are denoted here by *c*_*i*_, i.e. hiopt=N〈h2〉ci
 MathType@MTEF@5@5@+=feaafiart1ev1aaatCvAUfKttLearuWrP9MDH5MBPbIqV92AaeXatLxBI9gBamXvP5wqSXMqHnxAJn0BKvguHDwzZbqegyvzYrwyUfgarqqtubsr4rNCHbGeaGqiA8vkIkVAFgIELiFeLkFeLk=iY=Hhbbf9v8qqaqFr0xc9pk0xbba9q8WqFfeaY=biLkVcLq=JHqVepeea0=as0db9vqpepesP0xe9Fve9Fve9GapdbaqaaeGacaGaaiaabeqaamqadiabaaGcbaGaemiAaG2aa0baaSqaaiabdMgaPbqaaGWaaiaa=9gacaWFWbGaa8hDaaaakiabg2da9maakaaabaGaemOta4KaeyykJeUaemiAaG2aaWbaaSqabeaacqaIYaGmaaGccqGHQms8cqWGJbWydaWgaaWcbaGaemyAaKgabeaaaeqaaaaa@4DCD@.

However, selection in the SCN model is applied not only to the native energy, which estimates stability with respect to unfolding, but also to the stability against misfolded compact structures. The relevance of molecular diseases related to misfolding and aggregation [43], the importance for cell physiology and evolution of molecular chaperones preventing misfolding [44-46], and comparative analysis of thermodynamic properties of homologous proteins [39] all indicate that selection for stability against misfolding is an important selective force. In our model, this is achieved imposing a minimal allowed value for the normalized energy gap (see Methods).

Therefore, we set out to minimize the native energy with a large value of the normalized energy gap. The computation is reported in the Methods section. The implicit analytic solution, Eq. (17), receives its main contribution from the PE. This contribution is overwhelming in case of structures without internal modularity. Therefore, we further approximate the optimal HP as the sum of the PE profile plus a term which is constant at all sites. This is equivalent to stating that the correlation coefficient between PE and HP is one, and yields

hiopt≈〈h2〉−〈h2〉(〈c2〉−〈c〉2)(ci−〈c〉)+〈h〉.     (3)
 MathType@MTEF@5@5@+=feaafiart1ev1aaatCvAUfKttLearuWrP9MDH5MBPbIqV92AaeXatLxBI9gBaebbnrfifHhDYfgasaacH8akY=wiFfYdH8Gipec8Eeeu0xXdbba9frFj0=OqFfea0dXdd9vqai=hGuQ8kuc9pgc9s8qqaq=dirpe0xb9q8qiLsFr0=vr0=vr0dc8meaabaqaciaacaGaaeqabaqabeGadaaakeaacqWGObaAdaqhaaWcbaGaemyAaKgabaWexLMBbXgBcf2CPn2qVrwzqf2zLnharyqqYLwySbaceaGaa83Baiaa=bhacaWF0baaaOGaeyisIS7aaOaaaeaadaWcaaqaaiabgMYiHlabdIgaOnaaCaaaleqabaGaeGOmaidaaOGaeyOkJeVaeyOeI0IaeyykJeUaemiAaG2aaWbaaSqabeaacqaIYaGmaaGccqGHQms8aeaadaqadaqaaiabgMYiHlabdogaJnaaCaaaleqabaGaeGOmaidaaOGaeyOkJeVaeyOeI0IaeyykJeUaem4yamMaeyOkJe=aaWbaaSqabeaacqaIYaGmaaaakiaawIcacaGLPaaaaaaaleqaaOGaeiikaGIaem4yam2aaSbaaSqaaiabdMgaPbqabaGccqGHsislcqGHPms4cqWGJbWycqGHQms8cqGGPaqkcqGHRaWkcqGHPms4cqWGObaAcqGHQms8cqGGUaGlcaWLjaGaaCzcaiabcIcaOiabiodaZiabcMcaPaaa@6E46@

**Figure 1:**
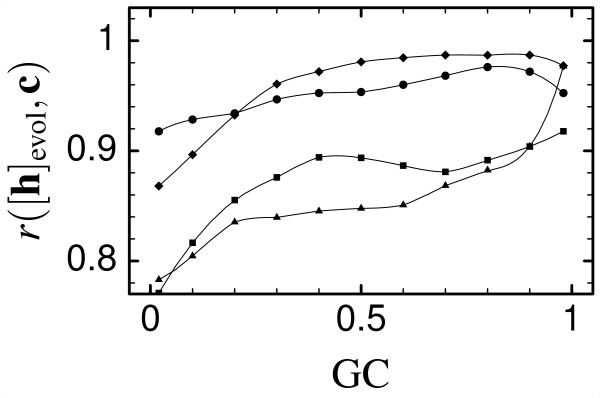
Correlation coefficient between the average HP and the PE for SCN simulations with various mutation models yielding different GC biases, for three single-domain proteins, lysozyme (PDB code 31zt, circles), phosphocarrier protein Hpr (PDB code 1opd, diamonds), and myoglobin (PDB code 1a6g, squares), and for the small two-domains protein ATP synthase *ε *unit (ATPE, PDB code 1aqt, triangles).

Before ending the section, we list the approximations involved in the above equation and its limits of validity. For comparison with simulation results, see Fig. 1 below.

1. We neglected the lower eigenvectors in the spectral expansion of the contact interaction matrix *U*(*a, b*). Simulations of the SCN model with equiprobable mutations show that this approximation is rather good, since one can get correlation coefficients larger than 0.95 between the simulated optimal HP and the PE of single-domain proteins, which would not be the case if the other components of the contact interaction matrix would pose significant constraints.

2. We neglected the discretization of the hydrophobicities in twenty values corresponding to the amino acids. This approximation is usually good for values of <*h*> and <*h*^2^> as observed in real proteins, which are much larger than the minimum and much smaller than the maximum of the twenty hydrophobicity values, but it is violated for extreme mutation bias.

3. We used the REM approximation for the normalized energy gap [47], which has been calculated through threading in the simulations. This is not a problem, since there is very good correlation between the REM estimate and the threading result at fixed sequence length. Moreover, since the threading calculation overestimates the normalized energy gap for long proteins, the REM approximation may even yield a more appropriate estimate [47].

4. We assumed that the lower eigenvectors contribute negligibly to the optimal hydrophobicity. This approximation is violated for multi-domain proteins, where other eigenvectors have non-negligible contributions. For single-domain proteins the corrections are usually small in the cases that we simulated.

### Evolutionary average of the hydrophobicity profile

In the SCN model, following Kimura's neutral model, all sequences having stability properties above some predetermined threshold are selectively equivalent. Therefore, the optimal sequence is very unlikely to be realized during evolution. However, all viable sequences must be sufficiently stable, which implies that they must have large correlation coefficient with the optimal HP. Thus, in SCN simulations protein sequences are expected to move around the optimal sequence, so that the evolutionary average of the HP almost coincides with the optimal HP [48]. This condition can be written formally as

[h(Ai)]evol≡∑{a}πi(a)h(a)=hiopt.     (4)
 MathType@MTEF@5@5@+=feaafiart1ev1aaatCvAUfKttLearuWrP9MDH5MBPbIqV92AaeXatLxBI9gBaebbnrfifHhDYfgasaacH8akY=wiFfYdH8Gipec8Eeeu0xXdbba9frFj0=OqFfea0dXdd9vqai=hGuQ8kuc9pgc9s8qqaq=dirpe0xb9q8qiLsFr0=vr0=vr0dc8meaabaqaciaacaGaaeqabaqabeGadaaakeaacqGGBbWwcqWGObaAcqGGOaakcqWGbbqqdaWgaaWcbaGaemyAaKgabeaakiabcMcaPiabc2faDnaaBaaaleaaieaacqWFLbqzcqWF2bGDcqWFVbWBcqWFSbaBaeqaaOGaeyyyIO7aaabuaeaaiiGacqGFapaCdaWgaaWcbaGaemyAaKgabeaakiabcIcaOiabdggaHjabcMcaPiabdIgaOjabcIcaOiabdggaHjabcMcaPiabg2da9iabdIgaOnaaDaaaleaacqWGPbqAaeaacqWFVbWBcqWFWbaCcqWF0baDaaGccqGGUaGlaSqaaiabcUha7jabdggaHjabc2ha9bqab0GaeyyeIuoakiaaxMaacaWLjaGaeiikaGIaeGinaqJaeiykaKcaaa@5A70@

The evolutionary average of the HP is indicated as [**h**]_evol _and it is expressed as a sum over all amino acids {*a*} of the site specific amino acid distribution at site *i *resulting from the evolutionary process, *π_i_*(*a*). The average sequence so defined is closely related to the prototype sequence defined by Bornberg-Bauer [21,22], which is maximally stable both thermodynamically and against mutations.

Combining Eq. (3) and (4), we obtain an analytic prediction of the average value of the site-specific amino acid distributions,

∑{a}πi(a)h(a)=〈h2〉−〈h2〉(〈c2〉−〈c〉2)(ci−〈c〉)+〈h〉.     (5)
 MathType@MTEF@5@5@+=feaafiart1ev1aaatCvAUfKttLearuWrP9MDH5MBPbIqV92AaeXatLxBI9gBaebbnrfifHhDYfgasaacH8akY=wiFfYdH8Gipec8Eeeu0xXdbba9frFj0=OqFfea0dXdd9vqai=hGuQ8kuc9pgc9s8qqaq=dirpe0xb9q8qiLsFr0=vr0=vr0dc8meaabaqaciaacaGaaeqabaqabeGadaaakeaadaaeqbqaaGGaciab=b8aWnaaBaaaleaacqWGPbqAaeqaaOGaeiikaGIaemyyaeMaeiykaKIaemiAaGMaeiikaGIaemyyaeMaeiykaKIaeyypa0ZaaOaaaeaadaWcaaqaaiabgMYiHlabdIgaOnaaCaaaleqabaGaeGOmaidaaOGaeyOkJeVaeyOeI0IaeyykJeUaemiAaG2aaWbaaSqabeaacqaIYaGmaaGccqGHQms8aeaadaqadaqaaiabgMYiHlabdogaJnaaCaaaleqabaGaeGOmaidaaOGaeyOkJeVaeyOeI0IaeyykJeUaem4yamMaeyOkJe=aaWbaaSqabeaacqaIYaGmaaaakiaawIcacaGLPaaaaaaaleqaaOGaeiikaGIaem4yam2aaSbaaSqaaiabdMgaPbqabaGccqGHsislcqGHPms4cqWGJbWycqGHQms8cqGGPaqkcqGHRaWkcqGHPms4cqWGObaAcqGHQms8cqGGUaGlcaWLjaGaaCzcaiabcIcaOiabiwda1iabcMcaPaWcbaGaei4EaSNaemyyaeMaeiyFa0habeqdcqGHris5aaaa@6F41@

The quantities <*h*> and <*h*^2^> are the only parameters that we need in order to compute the average HP at all sites of the protein as a function of the PE. In the following, these parameters will be measured in the simulations.

If the correlation coefficient between the PE and the average HP would be one, then the prediction Eq. (5) would be exact. We verified that the correlation coefficient is large (see below) and the analytic prediction yields a good fit of the average HP. The slope of the linear relationship between average HP and PE has a typical relative root mean square error of 9% with respect to the predicted value. The intercept has a typical relative error of 34%, but the mean absolute error is very small, 0.02, which is less than 5% of the mean HP. In the following, we further discuss the quality of the prediction reporting the correlation coefficient, which gives a strong indication of the relative error.

We plot in Fig. 1 the correlation coefficient between the average HP and the PE for SCN simulations with various mutation models characterized by different bias towards the nucleotides C+G, for lysozyme (PDB code 31zt), phosphocarrier protein Hpr (PDB code 1opd) and myoglobin (PDB code 1a6g), which are three single-domain proteins, and ATP synthase *ε *subunit (ATPE, PDB code 1aqt), which, despite its small size of 135 residues, has two domains, a small beta barrel and a two-helix bundle.

Except for extreme mutational bias the correlation coefficient between average HP and PE is always larger than 0.8. Lysozyme and Hpr have a similar behavior, characterized by high correlation coefficient between the average HP and the PE. The decrease of the correlation at extreme mutation bias is mainly due to the finite values of the minimal and maximal hydrophobicity. The same effect is present for myoglobin and ATPE, but for these proteins the correlation is lower because the contribution of other eigenvectors to the optimal HP is not negligible. Nevertheless, the correlation r([**h**]_evol_, **c**) is close to one for all four proteins for almost all the mutation models that we simulated. We found that a good predictor of the relevance of other eigenvectors is the quantity *η*_2 _defined in Eq. (20). This quantity is small (≈ 0.04) for the first two proteins and larger (≈ 0.4) for the other two and correlates strongly (*r *= -0.935) with *r*([**h**]_evol_, **c**) at zero GC bias (GC = 0.5)^2^. Since simulations of ATPE represents the worst case for our analytic theory, we will focus on them as an example in the rest of the paper.

### Effective selection model based on the principal eigenvector of the contact matrix

Our aim here is to exploit the results on the average HP in order to define an effective selection model that reproduces the SCN results based on explicit protein thermodynamics.

Let us first consider a mutation model where mutations from any amino acid to any other one are equiprobable. This is the mutation model that we simulated in our previous studies [29-31]. In this case, we assume that Eq. (5) is the only condition acting on the stationary amino acid distributions *π_i_*(*a*). This assumption is translated into the requirement that each *π_i_*(*a*) is the distribution of maximum entropy with mean values given by Eq. (5). As it is well known, this is an exponential, or Boltzmann, distribution of the form [49]

*π_i_*(*a*) ∝ exp [-*β_i _h*(*a*)],     (6)

The site-specific Boltzmann parameters *β_i _*can be computed analytically imposing that the average values ∑aπi(a)h(a)
 MathType@MTEF@5@5@+=feaafiart1ev1aaatCvAUfKttLearuWrP9MDH5MBPbIqV92AaeXatLxBI9gBaebbnrfifHhDYfgasaacH8akY=wiFfYdH8Gipec8Eeeu0xXdbba9frFj0=OqFfea0dXdd9vqai=hGuQ8kuc9pgc9s8qqaq=dirpe0xb9q8qiLsFr0=vr0=vr0dc8meaabaqaciaacaGaaeqabaqabeGadaaakeaadaaeqaqaaGGaciab=b8aWnaaBaaaleaaieGacqGFPbqAaeqaaOGaeiikaGIaemyyaeMaeiykaKIaemiAaGMaeiikaGIaemyyaeMaeiykaKcaleaacqWGHbqyaeqaniabggHiLdaaaa@3A88@ are given by Eq. (5), which depends only on the PE components *c*_*i *_and on the two parameters <*h*> and <*h*^2^>.

Details on the calculation of *β_i _*are given in the Methods section. *β_i _*takes both positive and negative values. Through the theorem of the implicit function it is easy to see that *β_i _*is a decreasing function of *c*_*i*_. This has a very simple interpretation: Positions with large *c*_*i *_are buried in the core of the protein, so they tend to have larger mean hydrophobicity, Eq. (5), and with higher probability they are occupied by hydrophobic amino acids, thus having more negative *β_i_*. Positions with small *c*_*i *_are exposed, tend to be hydrophilic, and have large and positive *β_i_*. This result is not surprising qualitatively, but it is remarkable that it allows to compute quantitatively the probability to observe a hydrophobic amino acid as a function of a structural indicator, the component *c*_*i *_of the principal eigenvector of the contact matrix, and on the two parameters <*h*> and <*h*^2^>.

Modelling amino acid distributions using Boltzmann distributions of physico-chemical properties had been previously proposed by Goldstein and coworkers [50,51] and by Shaknovich and coworkers [27,28]. Our approach differs from previous ones in the sense that we compute the Boltzmann parameter explicitly as a function of a structural indicator, the principal eigenvector of the contact matrix.

Several structural properties of proteins are found to follow Boltzmann distributions as well, i.e. the frequency of a structural motif is exponentially depending on its energy. For a review and a theoretical explanation based on the Random Energy Model, see [52].

The above result allows us to define a stochastic evolution process with independent sites that reproduces the site-specific distributions obtained through the SCN model where sites are interacting. The simplest site-specific transition matrices having Eq. (6) as its equilibrium distribution and satisfying detailed balance have the form

Psel(i)
 MathType@MTEF@5@5@+=feaafiart1ev1aaatCvAUfKttLearuWrP9MDH5MBPbIqV92AaeXatLxBI9gBaebbnrfifHhDYfgasaacH8akY=wiFfYdH8Gipec8Eeeu0xXdbba9frFj0=OqFfea0dXdd9vqai=hGuQ8kuc9pgc9s8qqaq=dirpe0xb9q8qiLsFr0=vr0=vr0dc8meaabaqaciaacaGaaeqabaqabeGadaaakeaacqWGqbaudaqhaaWcbaacbaGae83CamNae8xzauMae8hBaWgabaGae8hkaGccbiGae4xAaKMae8xkaKcaaaaa@352F@(*a, b*) = min {1, exp[-*β_i _*[*h*(*b*) - *h*(*a*)]]}.     (7)

It is possible to extend this selection model to take into account functional conservation, which is not considered in this paper.

### Mean-field model with selection and mutation

The condition on the site-specific average hydrophobicity, Eq. (5), is independent of the mutation model in our analytic approximation. Simulations show a weak dependence on the mutation parameters, see Fig. 1. For strong mutation bias, at very low GC, this dependence is a consequence of the fact that the average hydrophobicity approaches its maximum value. For intermediate bias, this dependence can be rationalized noting that the relevance of other eigenvectors depends on the parameter 1 - <*h*>/(N〈h2〉
 MathType@MTEF@5@5@+=feaafiart1ev1aaatCvAUfKttLearuWrP9MDH5MBPbIqV92AaeXatLxBI9gBaebbnrfifHhDYfgasaacH8akY=wiFfYdH8Gipec8Eeeu0xXdbba9frFj0=OqFfea0dXdd9vqai=hGuQ8kuc9pgc9s8qqaq=dirpe0xb9q8qiLsFr0=vr0=vr0dc8meaabaqaciaacaGaaeqabaqabeGadaaakeaadaGcaaqaceaa4zGaemOta4KaeyykJeUaemiAaG2aaWbaaSqabeaacqaIYaGmaaGccqGHQms8aSqabaaaaa@3472@<*c*>), which varies with the mutation bias. In any case, Eq. (5) is a good approximation to SCN simulations, except for extreme mutation bias.

The stochastic selection process described by Eq. (7) imposes that the average hydrophobicity follows Eq. (5). This effective selection process reproduces the site-specific amino acid distributions obtained through simulations of the SCN model with equiprobable mutation. Here we simulate the SCN model with a more realistic mutation process that takes into account the genetic code and represent mutations nucleotide level. The stochastic process that corresponds to this modified SCN model is the combination of two processes [53]: (1) A mutation process identical to the one simulated in the SCN model; (2) The selection process described by Eq. (7), which imposes that the site-specific average HP is perfectly correlated with the PE.

For implementing the nucleotide mutation model, we define the state of each site *i *as a codon **n **= {*n*_1_*n*_2_*n*_3_}. The substitution process is then decomposed into mutation and selection processes according to

P(i)(n,n′)≡PμCOD(n,n′)Psel(i)(A[n],A[n′])(n≠n′),     (8)
 MathType@MTEF@5@5@+=feaafiart1ev1aaatCvAUfKttLearuWrP9MDH5MBPbIqV92AaeXatLxBI9gBamrtHrhAL1wy0L2yHvtyaeHbnfgDOvwBHrxAJfwnaebbnrfifHhDYfgasaacH8akY=wiFfYdH8Gipec8Eeeu0xXdbba9frFj0=OqFfea0dXdd9vqai=hGuQ8kuc9pgc9s8qqaq=dirpe0xb9q8qiLsFr0=vr0=vr0dc8meaabaqaciaacaGaaeqabaWaaeGaeaaakeaafaqabeqacaaabaGaemiuaa1aaWbaaSqabeaacqGGOaakcqWGPbqAcqGGPaqkaaGccqGGOaakieqacqWFUbGBcqGGSaalcuWFUbGBgaqbaiabcMcaPiabggMi6kabdcfaqnaaDaaaleaaiiGacqGF8oqBaeaacqqGdbWqcqqGpbWtcqqGebaraaGccqGGOaakcqWFUbGBcqGGSaalcuWFUbGBgaqbaiabcMcaPiabdcfaqnaaDaaaleaacqqGZbWCcqqGLbqzcqqGSbaBaeaacqGGOaakcqWGPbqAcqGGPaqkaaGccqGGOaakimaacqqFaeFqcqGGBbWwcqWFUbGBcqGGDbqxcqGGSaalcqqFaeFqcqGGBbWwcuWFUbGBgaqbaiabc2faDjabcMcaPaqaaiabcIcaOiab=5gaUjabgcMi5kqb=5gaUzaafaGaeiykaKIaeiilaWIaaCzcaiaaxMaadaqadaqaaiabiIda4aGaayjkaiaawMcaaaaaaaa@6F1A@

where PμCOD
 MathType@MTEF@5@5@+=feaafiart1ev1aaatCvAUfKttLearuWrP9MDH5MBPbIqV92AaeXatLxBI9gBaebbnrfifHhDYfgasaacH8akY=wiFfYdH8Gipec8Eeeu0xXdbba9frFj0=OqFfea0dXdd9vqai=hGuQ8kuc9pgc9s8qqaq=dirpe0xb9q8qiLsFr0=vr0=vr0dc8meaabaqaciaacaGaaeqabaqabeGadaaakeaacqWGqbaudaqhaaWcbaacciGae8hVd0gabaacbaGae43qamKae43ta8Kae4hraqeaaaaa@3300@(**n**, **n**') is the codon mutation matrix arising from the mutation process at the nucleotide level (see Methods), the selection process is represented in Eq. (7), and A
 MathType@MTEF@5@5@+=feaafiart1ev1aaatCvAUfKttLearuWrP9MDH5MBPbIqV92AaeXatLxBI9gBamrtHrhAL1wy0L2yHvtyaeHbnfgDOvwBHrxAJfwnaebbnrfifHhDYfgasaacH8akY=wiFfYdH8Gipec8Eeeu0xXdbba9frFj0=OqFfea0dXdd9vqai=hGuQ8kuc9pgc9s8qqaq=dirpe0xb9q8qiLsFr0=vr0=vr0dc8meaabaqaciaacaGaaeqabaWaaeGaeaaakeaaimaacqWFaeFqaaa@3821@[**n**] represents the amino acid coded by the codon **n**. The diagonal elements are defined through the normalization condition

P(i)(n,n)=1−∑n′≠nPμCOD(n,n′)Psel(i)(A[n],A[n′]).     (9)
 MathType@MTEF@5@5@+=feaafiart1ev1aaatCvAUfKttLearuWrP9MDH5MBPbIqV92AaeXatLxBI9gBamrtHrhAL1wy0L2yHvtyaeHbnfgDOvwBHrxAJfwnamXvP5wqSXMqHnxAJn0BKvguHDwzZbqehyvzYrwyUfgarqqtubsr4rNCHbGeaGqiA8vkIkVAFgIELiFeLkFeLk=iY=Hhbbf9v8qqaqFr0xc9pk0xbba9q8WqFfeaY=biLkVcLq=JHqVepeea0=as0db9vqpepesP0xe9Fve9Fve9GapdbaqaaeGacaGaaiaabeqaamaaeiqbaaGcbaGaemiuaa1aaWbaaSqabeaacqGGOaakcqWGPbqAcqGGPaqkaaGccqGGOaakiqqacaWFUbGaeiilaWIaa8NBaiabcMcaPiabg2da9iabigdaXiabgkHiTmaaqafabaGaemiuaa1aa0baaSqaaGGaciab+X7aTbqaaiabboeadjabb+eapjabbseaebaaaeaaceWFUbGbauaacqGHGjsUcaWFUbaabeqdcqGHris5aOGaeiikaGIaa8NBaiabcYcaSiqa=5gagaqbaiabcMcaPiabdcfaqnaaDaaaleaacqqGZbWCcqqGLbqzcqqGSbaBaeaacqGGOaakcqWGPbqAcqGGPaqkaaGccqGGOaakimaacqqFaeFqcqGGBbWwcaWFUbGaeiyxa0LaeiilaWIae0haXhKaei4waSLab8NBayaafaGaeiyxa0LaeiykaKIaeiOla4IaaCzcaiaaxMaadaqadaqaaiabiMda5aGaayjkaiaawMcaaaaa@7D39@

We first assume that the mutation process at the DNA level satisfies detailed balance, also called reversibility in the molecular evolution literature. This means that the stationary nucleotide frequencies *f*(*n*) satisfy the equation *f*(*n*_1_)Pμnuc
 MathType@MTEF@5@5@+=feaafiart1ev1aaatCvAUfKttLearuWrP9MDH5MBPbIqV92AaeXatLxBI9gBaebbnrfifHhDYfgasaacH8akY=wiFfYdH8Gipec8Eeeu0xXdbba9frFj0=OqFfea0dXdd9vqai=hGuQ8kuc9pgc9s8qqaq=dirpe0xb9q8qiLsFr0=vr0=vr0dc8meaabaqaciaacaGaaeqabaqabeGadaaakeaacqWGqbaudaqhaaWcbaacciGae8hVd0gabaacbaGae4NBa4Mae4xDauNae43yamgaaaaa@33E0@(*n*_1_, *n*_2_) = *f*(*n*_2_)Pμnuc
 MathType@MTEF@5@5@+=feaafiart1ev1aaatCvAUfKttLearuWrP9MDH5MBPbIqV92AaeXatLxBI9gBaebbnrfifHhDYfgasaacH8akY=wiFfYdH8Gipec8Eeeu0xXdbba9frFj0=OqFfea0dXdd9vqai=hGuQ8kuc9pgc9s8qqaq=dirpe0xb9q8qiLsFr0=vr0=vr0dc8meaabaqaciaacaGaaeqabaqabeGadaaakeaacqWGqbaudaqhaaWcbaacciGae8hVd0gabaacbaGae4NBa4Mae4xDauNae43yamgaaaaa@33E0@(*n*_2_, *n*_1_), where Pμnuc
 MathType@MTEF@5@5@+=feaafiart1ev1aaatCvAUfKttLearuWrP9MDH5MBPbIqV92AaeXatLxBI9gBaebbnrfifHhDYfgasaacH8akY=wiFfYdH8Gipec8Eeeu0xXdbba9frFj0=OqFfea0dXdd9vqai=hGuQ8kuc9pgc9s8qqaq=dirpe0xb9q8qiLsFr0=vr0=vr0dc8meaabaqaciaacaGaaeqabaqabeGadaaakeaacqWGqbaudaqhaaWcbaacciGae8hVd0gabaacbaGae4NBa4Mae4xDauNae43yamgaaaaa@33E0@(*n*_1_, *n*_2_) is the mutation matrix at the nucleotide level.

Under this hypothesis, the stationary distribution for the full substitution process can be decomposed as the product of the amino acid frequency *w*_AA_(*a*) expected from the mutation process without selection, which is the same for all sites, times the site-specific distributions due to the selection process, Eq. (6),

*π_i_*(*a*) ∝ *w*_AA_(*a*) exp[-*β_i _h*(*a*)],     (10)

wAA(a)=∑nδ(a,A[n])wCOD(n).     (11)
 MathType@MTEF@5@5@+=feaafiart1ev1aaatCvAUfKttLearuWrP9MDH5MBPbIqV92AaeXatLxBI9gBamrtHrhAL1wy0L2yHvtyaeHbnfgDOvwBHrxAJfwnaebbnrfifHhDYfgasaacH8akY=wiFfYdH8Gipec8Eeeu0xXdbba9frFj0=OqFfea0dXdd9vqai=hGuQ8kuc9pgc9s8qqaq=dirpe0xb9q8qiLsFr0=vr0=vr0dc8meaabaqaciaacaGaaeqabaWaaeGaeaaakeaacqWG3bWDdaWgaaWcbaGaeeyqaeKaeeyqaeeabeaakiabcIcaOiabdggaHjabcMcaPiabg2da9maaqafabaacciGae8hTdqMaeiikaGIaemyyaeMaeiilaWccdaGae4haXhKaei4waSfcbeGae0NBa4Maeiyxa0LaeiykaKIaem4DaC3aaSbaaSqaaiabboeadjabb+eapjabbseaebqabaGccqGGOaakcqqFUbGBcqGGPaqkaSqaaiab95gaUbqab0GaeyyeIuoakiabc6caUiaaxMaacaWLjaWaaeWaaeaacqaIXaqmcqaIXaqmaiaawIcacaGLPaaaaaa@5A70@

The factor *w*_AA_(*a*) is obtained as the sum of the expected frequencies of its codons under mutation alone, *w*_COD_(**n**) = *f*(*n*_1_) *f*(*n*_2_) *f*(*n*_3_), with *n*_1_, *n*_2_, *n*_3 _the three nucleotides composing the codon **n**, and *f*(*n*) is the stationary frequency of nucleotide *n *under mutation alone. It is easy to see that the distribution Eq. (10) satisfies detailed balance with respect to the full substitution process, Eq. (8).

Eq. (6) is a special case of Eq. (10), with a constant mutation factor *w*_AA_(*a*) ≡ 1 for all amino acids, consistently with the assumption that all mutations are equiprobable. Combining Eq. (10) with Eq. (5), we obtain

∑{a}h(a)wAA(a)exp⁡[−βih(a)]∑{a}wAA(a)exp⁡[−βih(a)]=〈h2〉−〈h〉2(〈c2〉−〈c〉2)/〈c〉2(ci〈c〉−1)+〈h〉.     (12)
 MathType@MTEF@5@5@+=feaafiart1ev1aaatCvAUfKttLearuWrP9MDH5MBPbIqV92AaeXatLxBI9gBaebbnrfifHhDYfgasaacH8akY=wiFfYdH8Gipec8Eeeu0xXdbba9frFj0=OqFfea0dXdd9vqai=hGuQ8kuc9pgc9s8qqaq=dirpe0xb9q8qiLsFr0=vr0=vr0dc8meaabaqaciaacaGaaeqabaqabeGadaaakeaadaWcaaqaamaaqababaGaemiAaGMaeiikaGIaemyyaeMaeiykaKIaem4DaC3aaSbaaSqaaGqaaiab=feabjab=feabbqabaGccqGGOaakcqWGHbqycqGGPaqkcyGGLbqzcqGG4baEcqGGWbaCcqGGBbWwcqGHsisliiGacqGFYoGydaWgaaWcbaGaemyAaKgabeaakiabdIgaOjabcIcaOiabdggaHjabcMcaPiabc2faDbWcbaGaei4EaSNaemyyaeMaeiyFa0habeqdcqGHris5aaGcbaWaaabeaeaacqWG3bWDdaWgaaWcbaGae8xqaeKae8xqaeeabeaakiabcIcaOiabdggaHjabcMcaPiGbcwgaLjabcIha4jabcchaWjabcUfaBjabgkHiTiab+j7aInaaBaaaleaacqWGPbqAaeqaaOGaemiAaGMaeiikaGIaemyyaeMaeiykaKcaleaacqGG7bWEcqWGHbqycqGG9bqFaeqaniabggHiLdGccqGGDbqxaaGaeyypa0ZaaOaaaeaadaWcaaqaaiabgMYiHlabdIgaOnaaCaaaleqabaGaeGOmaidaaOGaeyOkJeVaeyOeI0IaeyykJeUaemiAaGMaeyOkJe=aaWbaaSqabeaacqaIYaGmaaaakeaadaqadaqaaiabgMYiHlabdogaJnaaCaaaleqabaGaeGOmaidaaOGaeyOkJeVaeyOeI0IaeyykJeUaem4yamMaeyOkJe=aaWbaaSqabeaacqaIYaGmaaaakiaawIcacaGLPaaacqGGVaWlcqGHPms4cqWGJbWycqGHQms8daahaaWcbeqaaiabikdaYaaaaaaabeaakmaabmaabaWaaSaaaeaacqWGJbWydaWgaaWcbaGaemyAaKgabeaaaOqaaiabgMYiHlabdogaJjabgQYiXdaacqGHsislcqaIXaqmaiaawIcacaGLPaaacqGHRaWkcqGHPms4cqWGObaAcqGHQms8cqGGUaGlcaWLjaGaaCzcaiabcIcaOiabigdaXiabikdaYiabcMcaPaaa@A359@

This equation is the central result of this work. It allows to compute the Boltzmann exponents *β_i_*, and from them the site-specific amino acid distributions as a function of the normalized PE of a protein structure, *c*_*i*_/<*c*>, which depend little on the protein length, of two hydrophobicity parameters, <*h*> and <*h*^2^>, and of the stationary frequencies *w*_AA_(*a*) of the mutation model, which must fulfill detailed balance. From this equation one sees that the Boltzmann exponents *β_i_*, which define the selection process, depend on the mutation factors *w*_AA_(*a*). This contrasts with the fact that in the SCN model mutation and selection are two independent processes. The entanglement between selection and mutation is a consequence of the mean-field approach: We reduce an evolutionary process where sites are interrelated to a process where sites evolve independently, but under the global constraint given by Eq. (5). Because of this global constraint, the evolutionary process becomes dependent on the average properties of the amino acid chain, which in turn depend on the mutation process. Hence, at the mean-field level, mutation and selection become interrelated.

### SCN model with genetic code

To test the mean-field model, the SCN model was simulated using several mutation schemes. In a first set of simulations, we used mutation matrices that fulfil detailed balance and depend on the stationary frequencies *f*(*n*), *n *∈ {A, T, C, G}, and on the transition-transversion ratio *k *(see Methods). We further imposed the conditions *f*(C) = *f*(G) and *f*(A) = *f*(T), called type 2 parity rule [54], so that there are only two parameters in the mutation matrix.

**Figure 2:**
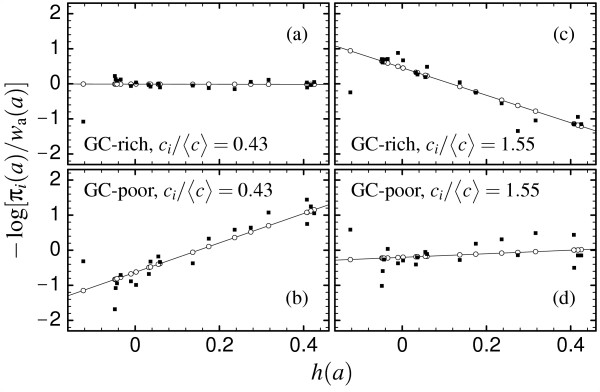
Comparison of the site-specific amino acid distribution *π_i_*(*a*) obtained from simulations of the SCN model for ATPE (PDB code 1aqt, full symbols) and from the mean-field model (lines and open symbols) at site *i *= 128 with *c*_*i*_/<*c*> = 0.43 [(a) and (b)] and at site *i *= 82 with *c*_*i*_/<*c*> = 1.55 [(c) and (d)]. The upper panels (a) and (c) show the case of high GC mutational bias, whereas the lower ones (b) and (d) show low GC mutational bias.

Fig. 2 shows the site-specific amino acid distributions obtained through SCN simulations for the protein ATPE (PDB code 1aqt), divided by the frequencies expected under mutation alone, *w*_AA_(*a*). The distributions plotted in Fig. 2(a) and 2(b) refer to a site with small PE component, *c*_*i*_/<*c*> = 0.43, favoring amino acids with low hydrophobicity. We show data for two different mutational biases, one favoring GC-rich codons (*f*(C) + *f*(G) = 0.8), which tend to be more hydrophylic, and one favoring GC-poor codons (*f*(C) + *f*(G) = 0.2), which tend to be more hydrophobic. The exponent *β_i _*changes with the mutational bias: For large GC bias the codons coding for hydrophilic amino acids are favored at the mutation level, and the *β_i _*is almost zero, meaning that almost no purifying selection is needed at this site. The contrary holds when the mutation bias favors GC-poor codons: In this case *β_i _*is large, and the site experiences strong purifying selection.

The opposite situation is observed in Fig. 2(c) and 2(d), obtained for a site with *c*_*i*_/<*c*> = 1.55. In this case amino acids with high hydrophobicity are preferred, and *β_i _*is almost zero when the mutational bias favors GC-poor codons, whereas it is negative and large when GC-rich codons are favored at the mutational level. In both cases, the mean-field model (straight lines and open symbols) fits very well the results of the SCN simulations (full symbols). The average over all sites of the correlation coefficients between predicted and observed amino acid distributions, for all mutational biases simulated, lies in the range <*r*> = 0.83 and <*r*> = 0.92 and increases as a function of the number of sequences examined (we simulated about 10^6 ^sequences for each mutational bias).

In all cases studied, the stationary amino acid distributions only depended on the stationary nucleotide frequencies *f*(*n*) and did not depend on the transition-transversion ratio, in agreement with Eq. (10). We also simulated a mutation process that does not fulfil detailed balance. In this case, Eq. (10) does not represent the stationary amino acid distribution of the process, which must be explicitly computed from the full transition matrix. Also in this case, Eq. (10), calculated with the stationary frequencies *f*(*n*) of the pure mutation process, is a very good approximation of the stationary distribution for high GC bias, but not for low GC, because of the large frequency of stop codons, which are strongly negatively selected (data not shown).

**Figure 3:**
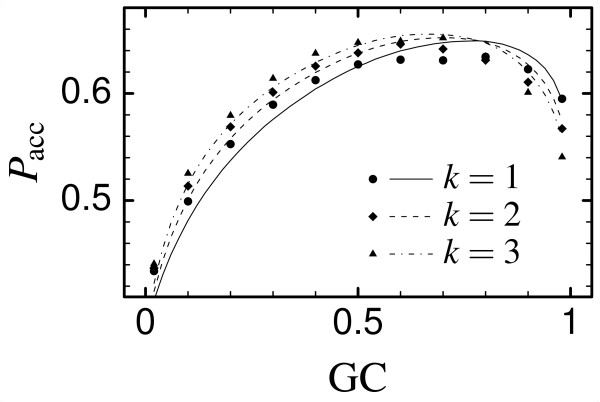
Acceptance probability for a mutation *P*_acc_, calculated in SCN simulations for ATPE (PDB code 1aqt, symbols) and in the mean-field model (lines) for three different values of the transition to transversion ratio *k *as a function of the GC content, *f*(C) + *f*(G). The mutation model is such that *P*(C) = *P*(G) and *P*(T) = *P*(A), assuming type 2 parity rule [54].

As a result of the change of the selection parameters *β_i _*with the mutation bias, the probability that a mutation is accepted depends on the mutation bias. This probability is also influenced by the transition-tranversion ratio *k*. The probability that a mutation is accepted, obtained from SCN simulations, and analytically computed from the effective stochastic process Eq. (8) (see Methods), is plotted in Fig. 3. The analytical calculation is in very good agreement with simulation results. Interestingly, the acceptance probability has a maximum as a function of the GC frequency, meaning that there is an optimal mutation bias that maximizes the rate of accepted mutations. This maximum is achieved at *f*(C) + *f*(G) > 0.5, since stop codons, which are the most deleterious mutations, are rich in AT. The acceptance probability increases with the transition to transversion ratio *k*, which favors more conservative mutations.

### Influence of the mutation bias on protein folding thermodynamics

In the SCN model, mutation and selection parameters influence the properties of protein folding thermodynamics arising from simulated evolution.

In both the SCN and the mean-field model, the nucleotide content at different codon positions responds differently to the mutation bias. The second position is the most difficult to mutate, whereas the third one is almost completely neutral, with GC_3 _≈ GC_mut_, since most transitions at third codon position are synonymous and they are always accepted in the SCN model. Thymine content at second codon position is least dependent on the mutation bias. Almost all codons containing T in the second position code for hydrophobic amino acids, so that the T content at second position is strongly correlated with the hydrophobicity of the coded protein and it is strongly constrained by selection for thermodynamic stability. A mutation bias towards T at the nucleotide level corresponds to a mutation bias towards more hydrophobic amino acids.

Also the selection thresholds on the native energy and the normalized energy gap influence the hydrophobicity. In fact, the more hydrophobic a protein is, the more stable it is against unfolding (it has lower effective energy), and the less stable it is against misfolding (it has smaller normalized energy gap). Stronger selection for a large normalized energy gap has thus the effect to reduce the hydrophobicity, and stronger selection for a low energy has the effect to increase it. The results presented here are obtained varying the mutation bias at constant selection thresholds, so that the differences between different simulations are due to mutation alone. We choose the selection thresholds equal to 98% of the values of the stability parameters in the PDB sequence. In this way, the PDB sequence is always selected, and we get stringent stability criteria that are as uniform as possible between different proteins.

Therefore, mutation and selection parameters modify the trade-off between the two kinds of stability and influence the mean and mean square hydrophobicity, <*h*> and <*h*^2^>. These quantities are used as parameters to calculate the site-specific distributions in the mean-field model. Unfortunately, we could not predict them analytically as a function of the mutation and selection parameters. The parameters used in the mean-field model were therefore derived from the sequences generated through SCN simulations. Nevertheless, using parameters <*h*> and <*h*^2^> that do not depend on the mutation bias in Eq. (5) still produces a good agreement between the mean-field and simulation results.

**Figure 4:**
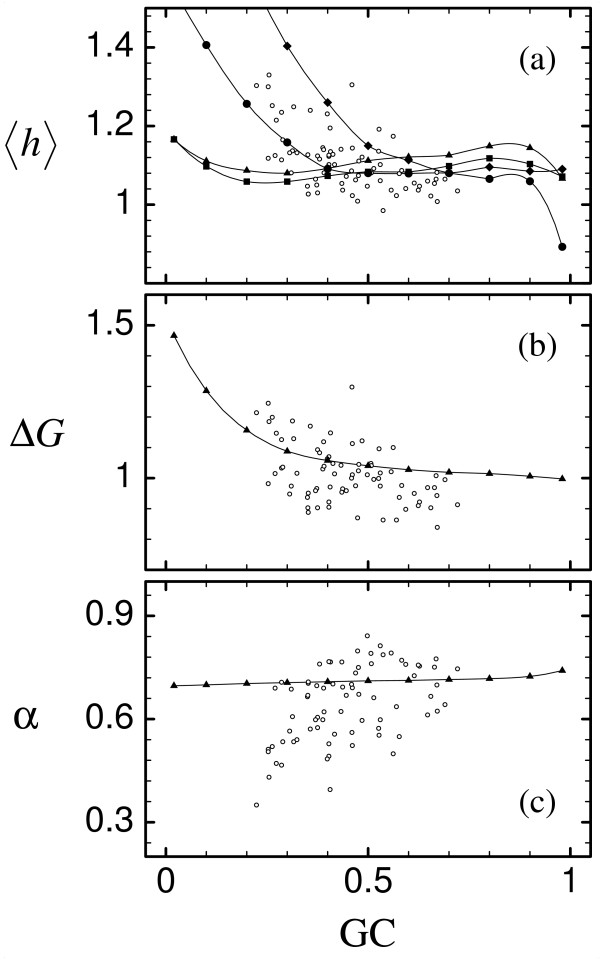
Full symbols and lines indicate average properties of protein folding thermodynamics in SCN simulations, open symbols indicate the same quantities in the proteomes of different bacterial species [39]. The horizontal axis represents the GC mutation bias for SCN simulations and the GC content at third codon position of the bacterial genes, (a) Mean hydrophobicity. SCN results are rescaled by a factor 8.6 and correspond to three single-domain proteins, lysozyme (PDB code 31zt, circles), phosphocarrier protein Hpr (PDB code 1opd, diamonds), and myoglobin (PDB code 1a6g, squares), and for the small two-domain protein ATP synthase *ε *unit (ATPE, PDB code 1aqt, triangles), (b) Mean unfolding free energy. SCN results are rescaled by a factor 4.3. Only ATPE is represented, the other proteins being qualitatively equivalent. (c) Mean normalized energy gap. SCN results are rescaled by a factor 1.3. Only ATPE is represented, the other proteins being qualitatively equivalent.

The dependence of hydrophobicity on the mutation bias has a deep influence on protein folding thermodynamics. For all the proteins that we simulated, the mean square hydrophobicity <*h*^2^> is a decreasing function of the G+C content (or, equivalently, an increasing function of the A+T content), causing the normalized energy gap to increase and the unfolding free energy per residue to decrease for increasing GC. We observed this effect in our SCN simulations. The same qualitative influence of the mutation bias on protein folding thermodynamics was inferred through a statistical analysis of the properties of orthologous proteins in different bacterial species evolving with different mutation biases [39]. Fig. 4 shows the mean hydrophobicity <*h*>, the effective energy per residue -*E/N *and the normalized energy gap *α *as a function of the GC content. Full symbols and lines are derived from simulations of the SCN model and open symbols are derived from the computational study of bacterial proteomes mentioned above [39]. Both sets of points show a similar trend, but the dispersion of thermodynamic properties is much larger in the bacterial proteomes than in SCN simulations.

There is however a qualitative difference between real and simulated data concerning mean hydrophobicity. In real sequences there is a negative correlation between the GC content of the gene and the mean hydrophobicity (*r *= -0.57, *P *< 10^-4^), as expected from the fact that T in second position codes for hydrophobic residues. In simulated sequences the correlation between GC content and mean hydrophobicity is negative in the whole GC range, but it is positive in the range of biologically observed GC values for two of the proteins simulated, ATPE and myoglobin. For the other two proteins, lysozyme and Hpr, the correlation is always negative. In contrast, the root mean square hydrophobicity <*h*^2^> is a monotonically decreasing function of the GC for all four proteins (data not shown). Interestingly, the proteins showing a positive correlation between GC content and mean hydrophobicity are those for which other eigenvectors besides the PE are relevant. This suggests that the hydrophobicity has a uneven distribution between the different modules that correspond to the relevant eigenvectors, so that, at decreasing GC mutation pressure, the mean hydrophobicity increases in one module and decreases in the other one responding to selection for stability against misfolding. The net effect is the lowering of the mean hydrophobicity while increasing its within sequence variation. It is possible that the resulting positive correlation between GC and hydrophobicity is an artifact of the SCN model, but it is also possible that it is a property of modular proteins that was not detected in the study of bacterial proteomes, since in that study data from unimodular and from multi-modular proteins were averaged together. This point deserves therefore further investigation.

It should also be noted that the normalized energy gap *α *is much smaller in bacterial proteomes corresponding to genomes with low GC content than expected from SCN simulations. As discussed in Ref. [39], these proteomes with very low GC content belong to obligatory intracellular bacteria, whose effective population size is severely reduced by the bottlenecks that they experience in their intracellular lifestyle. Because of their reduced populations, natural selection is expected to be less effective in eliminating deleterious mutations, causing their phenotypic properties, such as the folding stability of their proteins, to be less stable than for large free living populations [55-57].

Fig. 4 also suggests that natural selection acts on different protein properties for different mutation bias. When the mutation bias favors GC, the normalized energy gap tend to be higher than the threshold and the unfolding free energy tend to be small. In this case, most of the mutations that are selected against are eliminated because they yield proteins too unstable against unfolding. On the contrary, when the mutation pressure favors AT, most of the mutations that are eliminated yield proteins that are unstable against misfolding.

Last, we note that, despite simulated and observed properties have a qualitatively similar response to the mutation bias, from a quantitative point of view simulated quantities depend much more strongly on the mutation parameters. In particular, the GC content at first and second codon position, which influences the nature of the coded amino acids, depends on the mutation bias much more strongly in simulated genes than in real genes (see the Discussion).

### Site-specific amino acid distributions in the PDB

The predicted optimal hydrophobicity vector depends on the two parameters <*h*> and <*h*^2^>, in particular through the ratio <*h*>/〈h2〉
 MathType@MTEF@5@5@+=feaafiart1ev1aaatCvAUfKttLearuWrP9MDH5MBPbIqV92AaeXatLxBI9gBaebbnrfifHhDYfgasaacH8akY=wiFfYdH8Gipec8Eeeu0xXdbba9frFj0=OqFfea0dXdd9vqai=hGuQ8kuc9pgc9s8qqaq=dirpe0xb9q8qiLsFr0=vr0=vr0dc8meaabaqaciaacaGaaeqabaqabeGadaaakeaadaGcaaqaaiabgMYiHlabdIgaOnaaCaaaleqabaGaeGOmaidaaOGaeyOkJepaleqaaaaa@32CC@, in such a way that the optimal HP is almost parallel to the PE when <*h*>/〈h2〉
 MathType@MTEF@5@5@+=feaafiart1ev1aaatCvAUfKttLearuWrP9MDH5MBPbIqV92AaeXatLxBI9gBaebbnrfifHhDYfgasaacH8akY=wiFfYdH8Gipec8Eeeu0xXdbba9frFj0=OqFfea0dXdd9vqai=hGuQ8kuc9pgc9s8qqaq=dirpe0xb9q8qiLsFr0=vr0=vr0dc8meaabaqaciaacaGaaeqabaqabeGadaaakeaadaGcaaqaaiabgMYiHlabdIgaOnaaCaaaleqabaGaeGOmaidaaOGaeyOkJepaleqaaaaa@32CC@ is almost equal to N
 MathType@MTEF@5@5@+=feaafiart1ev1aaatCvAUfKttLearuWrP9MDH5MBPbIqV92AaeXatLxBI9gBaebbnrfifHhDYfgasaacH8akY=wiFfYdH8Gipec8Eeeu0xXdbba9frFj0=OqFfea0dXdd9vqai=hGuQ8kuc9pgc9s8qqaq=dirpe0xb9q8qiLsFr0=vr0=vr0dc8meaabaqaciaacaGaaeqabaqabeGadaaakeaadaGcaaqaaiabd6eaobWcbeaaaaa@2DEC@<*c*> (see Methods). Before applying our model to real proteins, we measured these quantities in a non-redundant subset of the Protein Data Bank (PDB) [58], containing both single-domain and multi-domain proteins, filtered to select only globular proteins.

**Figure 5:**
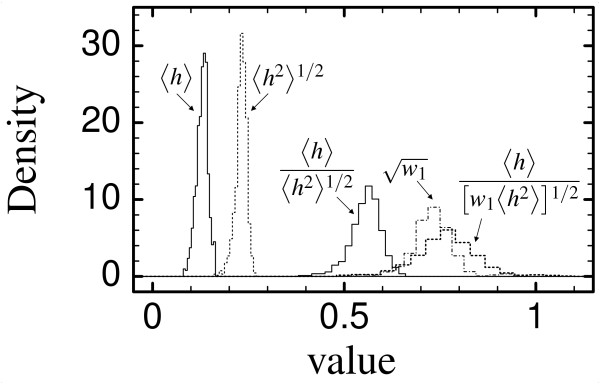
Distributions of hydrophobicity related quantities from a non-redundant subset of the PDB: Mean hydrophobicity; Root mean square hydrophobicity; Ratio between mean and root mean square hydrophobicity, *τ*, ratio between *τ *= <*h*>/〈h2〉
 MathType@MTEF@5@5@+=feaafiart1ev1aaatCvAUfKttLearuWrP9MDH5MBPbIqV92AaeXatLxBI9gBaebbnrfifHhDYfgasaacH8akY=wiFfYdH8Gipec8Eeeu0xXdbba9frFj0=OqFfea0dXdd9vqai=hGuQ8kuc9pgc9s8qqaq=dirpe0xb9q8qiLsFr0=vr0=vr0dc8meaabaqaciaacaGaaeqabaqabeGadaaakeaadaGcaaqaaiabgMYiHlabdIgaOnaaCaaaleqabaGaeGOmaidaaOGaeyOkJepaleqaaaaa@32CC@ and W1
 MathType@MTEF@5@5@+=feaafiart1ev1aaatCvAUfKttLearuWrP9MDH5MBPbIqV92AaeXatLxBI9gBaebbnrfifHhDYfgasaacH8srps0lbbf9q8WrFfeuY=Hhbbf9v8qqaqFr0xc9pk0xbba9q8WqFfea0=yr0RYxir=Jbba9q8aq0=yq=He9q8qqQ8frFve9Fve9Ff0dmeaabaqaciaacaGaaeqabaqabeGadaaakeaadaGcaaqcaawaaiabdEfaxPWaaSbaaKqaGfaacqaIXaqmaeqaaaqabaaaaa@2F29@ = N
 MathType@MTEF@5@5@+=feaafiart1ev1aaatCvAUfKttLearuWrP9MDH5MBPbIqV92AaeXatLxBI9gBaebbnrfifHhDYfgasaacH8akY=wiFfYdH8Gipec8Eeeu0xXdbba9frFj0=OqFfea0dXdd9vqai=hGuQ8kuc9pgc9s8qqaq=dirpe0xb9q8qiLsFr0=vr0=vr0dc8meaabaqaciaacaGaaeqabaqabeGadaaakeaadaGcaaqaaiabd6eaobWcbeaaaaa@2DEC@<*c*>. All these quantities are narrowly distributed, with standard deviations of the order of less than 1/10 of the average value.

Protein sequences in this set have <*h*> and <*h*^2^> contained in a narrow range, with standard deviation equal to 1/10 of the average value or smaller. Correspondingly, *τ *= <*h*>/〈h2〉
 MathType@MTEF@5@5@+=feaafiart1ev1aaatCvAUfKttLearuWrP9MDH5MBPbIqV92AaeXatLxBI9gBaebbnrfifHhDYfgasaacH8akY=wiFfYdH8Gipec8Eeeu0xXdbba9frFj0=OqFfea0dXdd9vqai=hGuQ8kuc9pgc9s8qqaq=dirpe0xb9q8qiLsFr0=vr0=vr0dc8meaabaqaciaacaGaaeqabaqabeGadaaakeaadaGcaaqaaiabgMYiHlabdIgaOnaaCaaaleqabaGaeGOmaidaaOGaeyOkJepaleqaaaaa@32CC@ lies in a narrow range between 0.4 and 0.65 (mean value 0.56), and *τ*/N
 MathType@MTEF@5@5@+=feaafiart1ev1aaatCvAUfKttLearuWrP9MDH5MBPbIqV92AaeXatLxBI9gBaebbnrfifHhDYfgasaacH8akY=wiFfYdH8Gipec8Eeeu0xXdbba9frFj0=OqFfea0dXdd9vqai=hGuQ8kuc9pgc9s8qqaq=dirpe0xb9q8qiLsFr0=vr0=vr0dc8meaabaqaciaacaGaaeqabaqabeGadaaakeaadaGcaaqaaiabd6eaobWcbeaaaaa@2DEC@<*c*> lies in a range between 0.5 and 1.1 (mean value 0.77). The largest values belong to multi-domain proteins, whose N
 MathType@MTEF@5@5@+=feaafiart1ev1aaatCvAUfKttLearuWrP9MDH5MBPbIqV92AaeXatLxBI9gBaebbnrfifHhDYfgasaacH8akY=wiFfYdH8Gipec8Eeeu0xXdbba9frFj0=OqFfea0dXdd9vqai=hGuQ8kuc9pgc9s8qqaq=dirpe0xb9q8qiLsFr0=vr0=vr0dc8meaabaqaciaacaGaaeqabaqabeGadaaakeaadaGcaaqaaiabd6eaobWcbeaaaaa@2DEC@<*c*> is much smaller than for single-domain proteins. The values of *τ*/w1
 MathType@MTEF@5@5@+=feaafiart1ev1aaatCvAUfKttLearuWrP9MDH5MBPbIqV92AaeXatLxBI9gBaebbnrfifHhDYfgasaacH8akY=wiFfYdH8Gipec8Eeeu0xXdbba9frFj0=OqFfea0dXdd9vqai=hGuQ8kuc9pgc9s8qqaq=dirpe0xb9q8qiLsFr0=vr0=vr0dc8meaabaqaciaacaGaaeqabaqabeGadaaakeaadaGcaaqaaiabdEha3naaBaaaleaacqaIXaqmaeqaaaqabaaaaa@2F4F@ are close to one, thus supporting our approximation to neglect eigenvectors other than the PE in the computation of the average HP. The distributions are plotted in Fig. 5, which also shows the distribution of w1
 MathType@MTEF@5@5@+=feaafiart1ev1aaatCvAUfKttLearuWrP9MDH5MBPbIqV92AaeXatLxBI9gBaebbnrfifHhDYfgasaacH8akY=wiFfYdH8Gipec8Eeeu0xXdbba9frFj0=OqFfea0dXdd9vqai=hGuQ8kuc9pgc9s8qqaq=dirpe0xb9q8qiLsFr0=vr0=vr0dc8meaabaqaciaacaGaaeqabaqabeGadaaakeaadaGcaaqaaiabdEha3naaBaaaleaacqaIXaqmaeqaaaqabaaaaa@2F4F@ = N
 MathType@MTEF@5@5@+=feaafiart1ev1aaatCvAUfKttLearuWrP9MDH5MBPbIqV92AaeXatLxBI9gBaebbnrfifHhDYfgasaacH8akY=wiFfYdH8Gipec8Eeeu0xXdbba9frFj0=OqFfea0dXdd9vqai=hGuQ8kuc9pgc9s8qqaq=dirpe0xb9q8qiLsFr0=vr0=vr0dc8meaabaqaciaacaGaaeqabaqabeGadaaakeaadaGcaaqaaiabd6eaobWcbeaaaaa@2DEC@<*c*>.

In the following, we will consider only single-domain globular proteins (see Methods) and assume that their optimal HP is well approximated by Eq. (5), with the same parameters <*h*> and <*h*^2^> for all proteins. This is justified by the narrow distribution of these quantities. In this way, we are able to predict the average hydrophobicity for structurally equivalent positions, having the same value of *c*_*i*_/<*c*>, in all structures in the PDB, using only two parameters.

We sampled amino acid distributions at site classes characterized by the same value (within a narrow range) of *c*_*i*_/<*c*>). These observed site-specific distributions were then compared with the distributions arising from the mean-field model with different mutation schemes.

The agreement between predicted and observed distributions was measured through their mean correlation coefficient <*r*>. The parameters of the mutation models were fitted optimizing this quantity (see Methods). Note that only the mutation parameters were fitted, whereas the selection parameters *β_i _*were calculated from the mutation model and the site-specific mean hydrophobicities given by Eq. (5), which only involves the two parameters *A *and *B*. These were calculated from the mean and the standard deviation of the hydrophobicity in the whole set of globular proteins, without any fitting procedure.

We performed the computations described here using several hydrophobicity scales, listed in Methods. For all mutation models considered, the best fit was always obtained with the IH hydrophobicity scale [48], closely followed by the CH scale [48] and the buriability scale [59]. All other scales gave considerably worse results. In the following we refer to the IH scale when not otherwise stated.

We considered the following five mutation and selection schemes:

(1) No selection (*β_i _*≡ 0), nucleotide mutation matrices satisfying detailed balance with the equilibrium frequencies as free parameters. This scheme is labeled as 'opt. freq. at *β_i _*≡ 0'. Because of the normalization condition, there are only three free parameters. The optimal equilibrium frequencies are 0.265, 0.327, 0.175, and 0.233 (T, A, C, and G), and yield <*r*> = 0.56.

(2) Selection and uniform mutation probabilities at the amino acid level *P*^(*i*) ^(*a*, *b*) ≡ 1 without the genetic code being taken into account, i.e. *w*_AA_(*a*) ≡ 1. This scheme is labeled as 'constant'. For this case we obtained <*r*> = 0.70 without any free parameter. It appears therefore that properly considering the selection process in the mean-field model gives better results than taking into account the genetic code but disregarding the amino acid properties (hydrophobicity), as in scheme (1).

(3) Independent and identical nucleotide mutation matrices satisfying detailed balance with equal equilibrium frequencies for all nucleotides, with selection from the mean-field model. This scheme is labeled as '#codons', since it holds *w*_AA_(*a*) = number of codons. The nucleotide frequencies are 0.25, 0, 25, 0.25, and 0.25 (T, A, C, and G). In this case, both the genetic code and the selection process are considered, and we obtained <*r*> = 0.80, again without any free parameter.

(4) Independent and identical nucleotide mutation matrices satisfying detailed balance with equilibrium frequencies as free parameters, and selection taken from the mean-field model. This scheme is labeled as 'opt. freq.'. The optimal nucleotide frequencies are 0.243, 0.312, 0.189, and 0.257 (T, A, C, and G) and yield <*r*> = 0.86 with three free parameters.

(5) Same scheme as above, with an additional free parameter that expresses the enhancement of the mutation rate at CpG dinucleotides. Notice that this mutation scheme does not fulfil detailed balance, and mutations at different sites in the DNA sequences are not anymore independent. We only considered CpG dinucleotides within the same codon, otherwise the resulting mean-field model would not be anymore independent at different positions along the protein sequence. We label this scheme as 'CpG'. The optimal nucleotide frequencies are 0.193, 0.316, 0.210, and 0.281 (T, A, C, and G), and the enhancement of the mutation rate at CpG is *k*_CpG _= 5.6. These optimal parameters yield <*r*> = 0.90.

**Figure 6:**
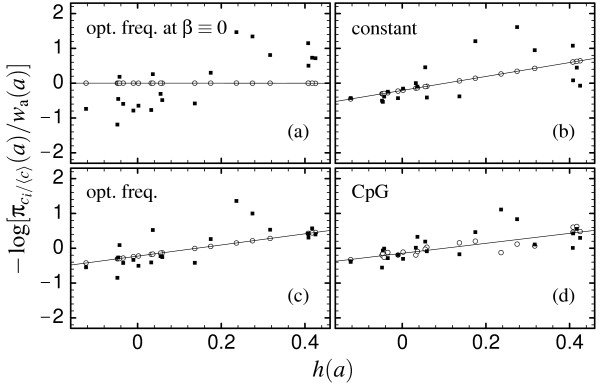
Observed and predicted site-specific amino acid distribution πci/<c>
 MathType@MTEF@5@5@+=feaafiart1ev1aaatCvAUfKttLearuWrP9MDH5MBPbIqV92AaeXatLxBI9gBamXvP5wqSXMqHnxAJn0BKvguHDwzZbqegyvzYrwyUfgarqqtubsr4rNCHbGeaGqiA8vkIkVAFgIELiFeLkFeLk=iY=Hhbbf9v8qqaqFr0xc9pk0xbba9q8WqFfeaY=biLkVcLq=JHqVepeea0=as0db9vqpepesP0xe9Fve9Fve9GapdbaqaaeGacaGaaiaabeqaamqadiabaaGcbaacciGae8hWda3aaSbaaSqaaiabdogaJnaaBaaameaacqWGPbqAcqGGVaWlcqGH8aapcqWGJbWycqGH+aGpaeqaaaWcbeaaaaa@45FE@(*a*), divided by the expected frequencies under mutation alone *w*_AA_(*a*), for (a) the mutation models 1 ('opt. freq. at β ≡ 0'), (b) mutation model 2 ('constant'), (c) mutation model 4 ('opt. freq.'), and (d) mutation model 5 ('CpG'). For the theoretical models where mutation satisfies detailed balance, πci
 MathType@MTEF@5@5@+=feaafiart1ev1aaatCvAUfKttLearuWrP9MDH5MBPbIqV92AaeXatLxBI9gBaebbnrfifHhDYfgasaacH8akY=wiFfYdH8Gipec8Eeeu0xXdbba9frFj0=OqFfea0dXdd9vqai=hGuQ8kuc9pgc9s8qqaq=dirpe0xb9q8qiLsFr0=vr0=vr0dc8meaabaqaciaacaGaaeqabaqabeGadaaakeaaiiGacqWFapaCdaWgaaWcbaGaem4yam2aaSbaaWqaaiabdMgaPbqabaaaleqaaaaa@317E@(a)/*w*_AA_(*a*) ∝ exp [-*β_i _h*(*a*)], so that the slope of the plot represents *β_i _*at this site class. For illustration, site class with *c_i_*/<*c*> ∈ [0.435, 0.545] was selected. Full symbols show the observed distributions obtained from sequences in the PDB, whereas the open symbols and the lines display the mean-field model.

Fig. 6 shows one example of observed and predicted site-specific amino acid distributions. For illustration, we chose the class of sites with *c*_*i*_/<*c*> *∈ *[0.435,0.545]. These are sites with small PE component, favoring amino acids with low hydrophobicity. Predictions (empty circles) were derived from the mean-field model with various mutation schemes (1, 2, 4 and 5, in the numeration above) and parameters that optimally fit the observed distributions at all site classes. Observed distributions sampled from protein sequences in the PDB are shown as full circles.

In order to make the plot more illustrative, the amino acid frequencies, both predicted and observed, were divided by the frequencies expected under mutation alone, *w*_AA_(*a*), which depend on the mutation model considered. In this way, for the mean-field models with mutation process satisfying detailed balance (1, 2 and 4), the plot represents in logarithmic scale the selection factor *Z*^-1^exp [-*β_i _h*(*a*)] (*Z *is a normalization constant). Since the horizontal scale represents the amino acid hydrophobicity *h*(*a*), one can directly see from the slope of the plot the Boltzmann factor *β_i _*and notice that it indeed depends on the mutation model considered. The mutation scheme 5 ('CpG', Fig. 6 bottom right) does not obey detailed balance, therefore the mean-field predictions, log (πci/〈c〉pred
 MathType@MTEF@5@5@+=feaafiart1ev1aaatCvAUfKttLearuWrP9MDH5MBPbIqV92AaeXatLxBI9gBaebbnrfifHhDYfgasaacH8akY=wiFfYdH8Gipec8Eeeu0xXdbba9frFj0=OqFfea0dXdd9vqai=hGuQ8kuc9pgc9s8qqaq=dirpe0xb9q8qiLsFr0=vr0=vr0dc8meaabaqaciaacaGaaeqabaqabeGadaaakeaaiiGacqWFapaCdaqhaaWcbaGaem4yam2aaSbaaWqaaiabdMgaPbqabaWccqGGVaWlcqGHPms4cqWGJbWycqGHQms8aeaaieaacqGFWbaCcqGFYbGCcqGFLbqzcqGFKbazaaaaaa@3CA6@(*a*)/*w*_AA_(*a*)) (open circles), do not lay on a straight line as a function of *h*(*a*). Nevertheless, Eq. (10), represented as a line in the figure, is still a good approximation for the mean-field amino acid distribution.

**Figure 7:**
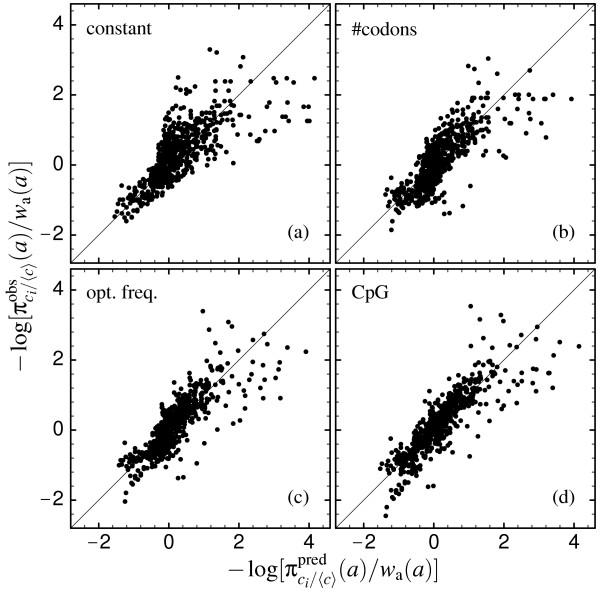
Site-specific amino acid frequencies sampled from the PDB, πci/〈c〉obs
 MathType@MTEF@5@5@+=feaafiart1ev1aaatCvAUfKttLearuWrP9MDH5MBPbIqV92AaeXatLxBI9gBaebbnrfifHhDYfgasaacH8akY=wiFfYdH8Gipec8Eeeu0xXdbba9frFj0=OqFfea0dXdd9vqai=hGuQ8kuc9pgc9s8qqaq=dirpe0xb9q8qiLsFr0=vr0=vr0dc8meaabaqaciaacaGaaeqabaqabeGadaaakeaaiiGacqWFapaCdaqhaaWcbaGaem4yam2aaSbaaWqaaiabdMgaPbqabaWccqGGVaWlcqGHPms4cqWGJbWycqGHQms8aeaatCvAUfeBSjuyZL2yd9gzLbvyNv2CaeHbwvMCKfMBHbaceaGaa43Baiaa+jgacaGFZbaaaaaa@4486@(*a*), versus the probabilities πci/〈c〉pred
 MathType@MTEF@5@5@+=feaafiart1ev1aaatCvAUfKttLearuWrP9MDH5MBPbIqV92AaeXatLxBI9gBaebbnrfifHhDYfgasaacH8akY=wiFfYdH8Gipec8Eeeu0xXdbba9frFj0=OqFfea0dXdd9vqai=hGuQ8kuc9pgc9s8qqaq=dirpe0xb9q8qiLsFr0=vr0=vr0dc8meaabaqaciaacaGaaeqabaqabeGadaaakeaaiiGacqWFapaCdaqhaaWcbaGaem4yam2aaSbaaWqaaiabdMgaPbqabaWccqGGVaWlcqGHPms4cqWGJbWycqGHQms8aeaaieaacqGFWbaCcqGFYbGCcqGFLbqzcqGFKbazaaaaaa@3CA6@(*a*) predicted through the mean-field model with optimal mutation parameters. All amino acids and all sites are shown. Observed and predicted frequencies are divided by the frequencies expected under mutation alone *w*_AA_(*a*). The four frames refer to (a) mutation model 2 ('constant'), (b) mutation model 3 ('#codons'), (c) mutation model 4 ('opt. freq.'), and (d) mutation model 5 ('CpG'), respectively.

In Fig. 7 we show the observed frequencies πci/〈c〉obs
 MathType@MTEF@5@5@+=feaafiart1ev1aaatCvAUfKttLearuWrP9MDH5MBPbIqV92AaeXatLxBI9gBaebbnrfifHhDYfgasaacH8akY=wiFfYdH8Gipec8Eeeu0xXdbba9frFj0=OqFfea0dXdd9vqai=hGuQ8kuc9pgc9s8qqaq=dirpe0xb9q8qiLsFr0=vr0=vr0dc8meaabaqaciaacaGaaeqabaqabeGadaaakeaaiiGacqWFapaCdaqhaaWcbaGaem4yam2aaSbaaWqaaiabdMgaPbqabaWccqGGVaWlcqGHPms4cqWGJbWycqGHQms8aeaatCvAUfeBSjuyZL2yd9gzLbvyNv2CaeHbwvMCKfMBHbaceaGaa43Baiaa+jgacaGFZbaaaaaa@4486@(*a*) versus the probabilities πci/〈c〉pred
 MathType@MTEF@5@5@+=feaafiart1ev1aaatCvAUfKttLearuWrP9MDH5MBPbIqV92AaeXatLxBI9gBaebbnrfifHhDYfgasaacH8akY=wiFfYdH8Gipec8Eeeu0xXdbba9frFj0=OqFfea0dXdd9vqai=hGuQ8kuc9pgc9s8qqaq=dirpe0xb9q8qiLsFr0=vr0=vr0dc8meaabaqaciaacaGaaeqabaqabeGadaaakeaaiiGacqWFapaCdaqhaaWcbaGaem4yam2aaSbaaWqaaiabdMgaPbqabaWccqGGVaWlcqGHPms4cqWGJbWycqGHQms8aeaaieaacqGFWbaCcqGFYbGCcqGFLbqzcqGFKbazaaaaaa@3CA6@(*a*) predicted through the mean-field model with optimal mutation parameters. All amino acid types and all sites are represented and, as in the previous figure, all frequencies are divided by the expected frequencies under mutation alone *w*_AA_(*a*). The four frames refer to mutation schemes 2, 3, 4 and 5. For model 1, which is not shown, the predicted probabilities coincide with the mutation factors (i.e., πci/〈c〉pred
 MathType@MTEF@5@5@+=feaafiart1ev1aaatCvAUfKttLearuWrP9MDH5MBPbIqV92AaeXatLxBI9gBaebbnrfifHhDYfgasaacH8akY=wiFfYdH8Gipec8Eeeu0xXdbba9frFj0=OqFfea0dXdd9vqai=hGuQ8kuc9pgc9s8qqaq=dirpe0xb9q8qiLsFr0=vr0=vr0dc8meaabaqaciaacaGaaeqabaqabeGadaaakeaaiiGacqWFapaCdaqhaaWcbaGaem4yam2aaSbaaWqaaiabdMgaPbqabaWccqGGVaWlcqGHPms4cqWGJbWycqGHQms8aeaaieaacqGFWbaCcqGFYbGCcqGFLbqzcqGFKbazaaaaaa@3CA6@(*a*) ≡ *w*_AA_(*a*)), so their ratio, represented on the horizontal line, is always one, and all points would lie on a vertical line and would yield no correlation between observed and predicted data. The best fit is obtained for models 4 and 5.

## Discussion

### Approximation used

Our analytic theory is based on a model of protein folding thermodynamics where the contact energy is approximated through *E*(**C, A**) ≈ - ∑_*ij *_*C_ij_h*(*A_i_*)*h*(*A_j_*), and the twenty parameters *h*(*a*) can be associated with an effective hydrophobicity. The thermodynamically optimal sequence for this model, for a fixed structure **C **of a single-domain globular protein, is the sequence whose HP *h*(*A*_*i*_) has correlation coefficient close to one with the PE of the contact matrix **C**. This result also holds with very good approximation for models with contact interactions, where the contact interaction matrix *U*(*a, b*) is well approximated by its main eigenvector *h*(*a*). This is the case of most contact interaction matrices used in protein folding studies, as for instance the Miyazawa and Jernigan interaction matrix [60], and it is well known that the main eigenvector represents hydrophobicity [40,41]. For the case of the interaction matrix used in this study [38], the correlation between the matrix elements *U*(*a, b*) and *h*(*a*)*h*(*b*) is larger than 0.80, therefore contributions other than hydrophobicity are not completely negligible. However, for single-domain globular proteins and for mutation models that are not extremely biased, the average hydrophobicity profile observed in SCN simulations with full contact interaction matrix is practically undistinguishable from the optimal HP predicted on the basis of the reduced matrix *h*(*a*)*h*(*b*), which justifies our theory *a posteriori*. The parameters *h*(*a*) are obtained from the main eigenvector of the interaction matrix, therefore they should not be interpreted as hydrophobicity in a strict biochemical sense, since they also take into account other kinds of interactions. For instance, aromatic amino acids have very large *h*(*a*), in part due to the strength of the interactions between aromatic rings. This is perhaps why the hydrophobicity scale that we use performs, for the purpose of predicting site-specific amino acid distributions, better than empirical hydrophobicity scales.

There are other kinds of interactions that can not be well approximated in this simple contact scheme, such as electrostatic interactions or local propensities for secondary structures, although the latter could be easily incorporated in the present scheme, and we are working in this direction. The good agreement between predicted and observed distributions, however, indicates that the energy function used captures a major component of the forces stabilizing protein folding.

Using the approximate free energy function, Eq. (2), and a continuous approximation for the hydrophobicity values *h*(*A*_*i*_), it is possible to determine analytically the sequence with minimal energy subject to the constraint of constant normalized energy gap. The HP of this optimally stable sequence is given by Eq. (17). For single-domain globular proteins, this optimal HP is almost parallel to the main eigenvector of the contact matrix (the PE).

It may be useful to recall some properties of the PE, in order to clarify its interpretation. The PE is the vector *c*_*i *_which maximizes the quadratic form *Q *= ∑_*ij*_*C_ij_c_i_c_j _*for fixed value of the norm ∑*_i_*ci2
 MathType@MTEF@5@5@+=feaafiart1ev1aaatCvAUfKttLearuWrP9MDH5MBPbIqV92AaeXatLxBI9gBaebbnrfifHhDYfgasaacH8akY=wiFfYdH8Gipec8Eeeu0xXdbba9frFj0=OqFfea0dXdd9vqai=hGuQ8kuc9pgc9s8qqaq=dirpe0xb9q8qiLsFr0=vr0=vr0dc8meaabaqaciaacaGaaeqabaqabeGadaaakeaacqWGJbWydaqhaaWcbaGaemyAaKgabaGaeGOmaidaaaaa@3075@. Therefore, it can be interpreted as an effective connectivity, since positions *i *with large *c*_*i *_are in contact with as many as possible positions *j *with large *c*_*j*_. Indeed, *c*_*i *_correlates with the number of contacts formed by site *i*. Nevertheless, *c*_*i *_depends not only on the local contacts, but also on the global structure of the protein chain, for instance its modularity, and it gives a much richer information than the simple number of contacts. It has been shown that a detailed knowledge of the PE is sufficient to reconstruct the full contact matrix [61], whereas different contact matrices may be associated to the same contact vector [62], whose components are the number of contacts at each site.

### Influence of mutation on evolutionary and thermodynamic properties

It was observed several years ago that the nucleotide content at first and second codon positions, which influences the coded amino acid and the amino acid usage, is strongly correlated with the nucleotide content at third codon position, where transitions (A-G and T-C mutations) in most cases do not modify the coded amino acid [54,63]. As a consequence, the amino acid usage in proteins of different bacterial species, which may evolve with different mutation bias, is strongly dependent on the mean nucleotide content of the genome, which is thought to reflect essentially the mutation bias. However, this dependence is not as strong as one would predict from a model based on mutation alone [64], equivalent to our model 1 discussed in the Results section. This deviation from a pure mutation model reflects, at least in part, selection at the amino acid level.

SCN simulations of globular proteins, and the corresponding mean-field models, which take into account both mutation and selection, reproduce qualitatively these results. Our results show that the amino acid composition, and the GC content at first and second codon position, reflecting selection at the amino acid level, depend on the mutation bias and they are strongly correlated with the GC content at third codon position, but this correlation is weaker than one would expect under a mutation model alone.

SCN simulations also reveal the deep influence that the mutation process exerts on protein evolution. Selection for the stability of the native state has to fulfil two partially contrasting requirements: stability against unfolding and stability against compact misfolded conformations [39,47]. The mutation bias influences the balance between these two kinds of stabilities. For fixed selection parameters, mutation processes favoring GC rich codons favor protein sequences that are less hydrophobic, and which are predicted to be more stable against misfolded states but less stable against unfolding with respect to mutation processes favoring AT rich codons.

This trend of decreasing unfolding stability and increasing misfolding stability versus GC content occurs in SCN simulations for all the proteins that we studied, in agreement with a previous statistical analysis of bacterial proteomes [39]. However, for two of the proteins that we studied, the mean hydrophobicity was not a monotonous function of the GC content.

The mutation bias also influences very strongly the fraction of mutations that are eliminated by purifying selection. The optimum acceptance rate is observed for a slight bias towards GC. The nucleotide frequencies that optimize the match between the mean-field model and the observed distributions yield a nearly optimal acceptance rate.

However, in the SCN model the dependency between amino acid usage and mutation bias is significantly stronger than the one observed in the genes of different bacterial species coding for globular proteins. In particular, the GC content at the first and second codon position depends on the GC content at the third codon position more strongly in the SCN model than in bacterial genes. We discuss four possible explanations for this discrepancy, that indicate possible extensions of the model.

First, and most important in our opinion, the selection criterium used in SCN simulations is based on a contact free energy function, whose main contribution comes from the hydrophobicity effect and van der Waals interactions. Selection in protein evolution, on the other hand, also depends on functional constraints, on other stability constraints, as for instance secondary structure, and on constraints arising from protein dynamics. The ability of the mean-field model to reproduce site-specific amino acid distributions in the PDB, where these effects are averaged out, suggests that it captures important features of the selection process, but quite probably other selective forces are relevant as well.

Second, in the SCN simulations presented here we have considered mutation probabilities where we enforced the symmetry between nucleotides related through Watson and Crick pairing, *f*(A) = *f*(T) and *f*(G) = *f*(C), called type 2 parity rule [54]. The type 2 parity rule holds globally in a double-stranded DNA molecule. However, it is well known that the mutation process in the two DNA strands is different, leading to asymmetric strand composition called GC-skew [65]. Therefore, since the distribution of coding sequences in leading and lagging strands may be biased [65], there is no strict reason why this symmetry should hold for protein coding sequences, and in fact we found that the nucleotide contents under mutation alone that optimally fit the observed amino acid distributions do not follow parity rule 2. The results of the SCN model, such as for instance the C+G content at different codon positions, depend also on the proportion of C with respect to G and A with respect to T, and not only on the cumulative content of C+G. Therefore the results presented here, obtained imposing the type 2 parity rule, are only indicative of a qualitative trend and can not be compared quantitatively with bacterial genes coding for globular proteins.

As a third possible discrepancy between SCN simulations and biological data, we have calculated stationary amino acid distributions when the substituion process has reached equilibrium. However, in SCN simulations even for short proteins equilibrium is attained after a very long transient phase, of the order of 10^5 ^substitutions. This number of substitutions is far larger than the estimated number of substitutions that took place since the split of the major domains of life [66]. In a recent study, Jordan *et al*. [66] have proposed that the substitution process in proteins has not yet attained equilibrium, and that one can still find the fingerprint of ancestral amino acid distributions by looking at the present day evolutionary process. Last, we recall that the SCN simulations reported here have been performed in the rare mutation regime where the product of population size times mutation rate is *Mμ *≪ 1. In this regime, the population is genetically homogeneous. If this hypothesis does not hold, one observes a trend towards increased mutational robustness for increasing *Mμ *[33-35]. However, this "selection" for robustness, without any explicit selective force, is expected to result in an increased correlation between the HP of selected sequences and the optimal HP. In fact, it has been observed by Bornberg-Bauer in simulations of neutral protein evolution that one can identify a prototype sequence that is maximally stable both thermodynamically and mutationally [21,22]. In our model, the prototype sequence coincides with the sequence with the optimal HP, which is strongly correlated with the PE. Consistently, the mutational robustness increases as the HP of the sequence gets closer to the optimal HP, predicted through the PE, as we have verified in previous simulations [67]. Therefore, we do not expect that selection for mutational robustness modifies qualitatively the results presented here, as far as equilibrium properties are concerned.

### Amino acid distributions in the PDB

The mean-field model developed in this paper gives a satisfactory fit of the site-specific amino acid distributions observed in single-domain globular proteins. In applying the model to a representative subset of the PDB, we assumed that: (a) Its selection parameters, <*h*> and <*h*^2^>, are roughly the same for all proteins. This hypothesis is reasonable, since both quantity are narrowly distributed, with standard deviations smaller than 1/10 of the average value. (b) The mutation process is the same for all the genes from which PDB proteins are derived. This hypothesis is clearly not valid, since mutation patterns depend on the organism considered and, within the same organism, they depend on the DNA strand and on the distance from the origin of replication in bacterial genomes [65], and they are thought to vary broadly within the large eukaryotic genomes.

Nevertheless, the fact that our model fits well the observed distributions may suggest the existence of a general mutational pattern valid for different organisms. Freeman and coworkers [68] found a strong correlation between excess of coding sequences and excess of purine basis (i.e., frequency of G+A) in bacterial and, later, eukaryotic genomes. This pattern agrees quite well with the frequencies that optimal fit our models to the observed distributions, which are *f*(T) = 0.243, *f*(A) = 0.312, *f*(C) = 0.189, *f*(G) = 0.257 if the nucleotide frequencies are the only free parameters and detailed balance is assumed, and *f*(T) = 0.193, *f*(A) = 0.316, *f*(C) = 0.210, *f*(G) = 0.281 if the mutation rate is enhanced at CpG dinucleotides. In both cases, we find that the frequency of A is larger than that of T and the frequency of G is larger than that of C, i.e. the fitted parameters suggest that there is purine excess in protein coding genes.

However, for some bacterial genomes the coding excess is better correlated with the excess of G+T (keto excess) than with the purine excess. This is consistent with the fact that in bacterial chromosomes there are two types of mutational patterns: (1) Predominance of G over C (positive GC skew) and of A over T (positive AT skew) in the leading strand, the opposite in the lagging strand, implying purine excess in coding sequences that are overrepresented in the leading strand; (2) Predominance of G over C (positive GC skew) and of T over A (negative AT skew) in the leading strand, the opposite in the lagging strand, implying keto excess in coding sequences [69]. In agreement with our SCN simulations, these mutational bias strongly influence the amino acid frequencies for proteins coded in the two strands [70]. The protein sequences that we studied in this work were derived from the PDB, where nucleotide sequences are not stored. We did not find any study of nucleotide frequencies in genes coding for proteins in the PDB. Therefore, we could not compare our fitted parameters to nucleotide frequencies in the genes coding for the proteins in the PDB. It would be interesting to know whether the purine excess that our study suggests is observed in these genes.

## Conclusion

We have shown that an evolutionary model with independently evolving sites is able to reproduce in a quantitative way the results of simulations where conservation of the thermodynamic stability of a protein native state is explicitly enforced, and therefore sites evolve in a correlated way. This mean-field model with independent sites is also able to reproduce site-specific amino acid distributions at sites with specific values of the principal eigenvector of the contact matrix, in good agreement with the distributions observed in the whole PDB. As site-independent evolutionary models are readily amenable to computation, we expect these results to be widely applicable in the context of the reconstruction of evolutionary histories. Our results also demonstrate that the mutation process has a deep influence on protein evolution. It modifies the balance between stability against the unfolded state and stability against misfolded compact conformations, and it modifies the fraction of mutations which are eliminated by purifying selection. In our mean-field model, mutations and selection are strictly interrelated, as the effective selection probabilities depend on the mutation process.

## Methods

### SCN model of neutral evolution

In the Structurally Constrained Neutral (SCN) model of protein evolution [29-32] amino acid mutations are proposed randomly, and accepted according to a stability criterion. The stability of the folded protein, is defined by an effective free energy function, *E*(*A, C*), based on contact interactions,

E(A,C)=∑i<jCijU(Ai,Aj),     (13)
 MathType@MTEF@5@5@+=feaafiart1ev1aaatCvAUfKttLearuWrP9MDH5MBPbIqV92AaeXatLxBI9gBaebbnrfifHhDYfgasaacH8akY=wiFfYdH8Gipec8Eeeu0xXdbba9frFj0=OqFfea0dXdd9vqai=hGuQ8kuc9pgc9s8qqaq=dirpe0xb9q8qiLsFr0=vr0=vr0dc8meaabaqaciaacaGaaeqabaqabeGadaaakeaacqWGfbqrcqGGOaakieqacqWFbbqqcqGGSaalcqWFdbWqcqWFPaqkcqWF9aqpdaaeqbqaaiabdoeadnaaBaaaleaacqWGPbqAcqWGQbGAaeqaaOGaemyvauLaeiikaGIaemyqae0aaSbaaSqaaiabdMgaPbqabaGccqGGSaalcqWGbbqqdaWgaaWcbaGaemOAaOgabeaakiabcMcaPaWcbaacbmGae4xAaKMae4hpaWJae4NAaOgabeqdcqGHris5aOGaeiilaWIaaCzcaiaaxMaacqGGOaakcqaIXaqmcqaIZaWmcqGGPaqkaaa@4C02@

where **A **represents the protein sequence, **C **is the contact map of the native structure, and **U **is a 20 × 20 symmetric matrix whose element *U*(*a, b*) represents the effective interaction, in units of *k*_B_*T*, of amino acids of types *a *and *b*; we use the interaction matrix derived by Bastolla *et al*. [38]. For most protein chains in the PDB this interaction matrix assigns lower effective free energy, Eq. (13), to the native structure than to decoys generated by threading, and it produces a well correlated free energy landscape. We then estimate two parameters: (i) The effective energy per residue, *E*(**A, C**)/*N*, Eq. (13), where *N *is the protein length. This quantity correlates with the folding free energy per residue for a set of 18 small proteins that are folding with two-states thermodynamics (correlation coefficient *r *= 0.91; UB, unpublished result); (ii) The normalized energy gap *α*, which characterizes fast folding sequences [71] with well correlated energy landscapes [17,72-75]. In the SCN model, mutated sequences are considered thermodynamically stable if both stability parameters are above predetermined thresholds. Synonymous mutations are always accepted, whereas mutations to stop codons are always rejected.

The normalized energy gap *α*(**A**), estimating misfolding stability, is defined as the minimal value of the difference between the energy of the native configuration, C*, and the energy of any compact configuration **C **satisfying the constraints of chain connectivity, excluded volume and hydrogen bonding, normalized times the absolute native energy and divided by the structural dissimilarity between the structures C* and **C**, 1 - *q*(C*,**C**), where *q *represents the contact overlap,

α(A)=min⁡CE(A,C)−E(A,C*)|E(A,C*)|[1−q(C,C*)].     (14)
 MathType@MTEF@5@5@+=feaafiart1ev1aaatCvAUfKttLearuWrP9MDH5MBPbIqV92AaeXatLxBI9gBaebbnrfifHhDYfgasaacH8akY=wiFfYdH8Gipec8Eeeu0xXdbba9frFj0=OqFfea0dXdd9vqai=hGuQ8kuc9pgc9s8qqaq=dirpe0xb9q8qiLsFr0=vr0=vr0dc8meaabaqaciaacaGaaeqabaqabeGadaaakeaaiiGacqWFXoqycqGGOaakieqacqGFbbqqcqGGPaqkcqGH9aqpdaWfqaqaaiGbc2gaTjabcMgaPjabc6gaUbWcbaGae43qameabeaakmaalaaabaGaemyrauKaeiikaGIae4xqaeKaeiilaWIae43qamKaeiykaKIaeyOeI0IaemyrauKaeiikaGIae4xqaeKaeiilaWIae43qamKaeiOkaOIaeiykaKcabaGaeiiFaWNaemyrauKaeiikaGIae4xqaeKaeiilaWIae43qamKaeiOkaOIaeiykaKIaeiiFaWNaei4waSLaeGymaeJaeyOeI0IaemyCaeNaeiikaGIae43qamKaeiilaWIae43qamKaeiOkaOIaeiykaKIaeiyxa0faaiabc6caUiaaxMaacaWLjaWaaeWaaeaacqaIXaqmcqaI0aanaiaawIcacaGLPaaaaaa@5F3E@

The normalized energy gap is estimated in our simulations from a set of alternative configurations. It depends strongly on the size of this set: We typically use hundreds of thousands of structures that can be generated by threading the protein sequence on all non-redundant structures in the PDB.

For the analytic calculation reported below, but not for the simulations, we use an estimate of the normalized energy gap [47] based on the Random Energy Model [76,77]

α(A)≈〈U〉A−σU,A2log⁡(mN)/Nc−E(A,C*)/Nc|E(A,C*)/Nc|(1−q0)     (15)
 MathType@MTEF@5@5@+=feaafiart1ev1aaatCvAUfKttLearuWrP9MDH5MBPbIqV92AaeXatLxBI9gBaebbnrfifHhDYfgasaacH8akY=wiFfYdH8Gipec8Eeeu0xXdbba9frFj0=OqFfea0dXdd9vqai=hGuQ8kuc9pgc9s8qqaq=dirpe0xb9q8qiLsFr0=vr0=vr0dc8meaabaqaciaacaGaaeqabaqabeGadaaakeaaiiGacqWFXoqycqGGOaakieqacqGFbbqqcqGGPaqkcqGHijYUdaWcaaqaaiabgMYiHlabbwfavjabgQYiXpaaBaaaleaacqGFbbqqaeqaaOGaeyOeI0Iaeq4Wdm3aaSbaaSqaaiabdwfavjabcYcaSiab+feabbqabaGcdaGcaaqaaiabikdaYiGbcYgaSjabc+gaVjabcEgaNjabcIcaOiabd2gaTnaaBaaaleaacqWGobGtaeqaaOGaeiykaKIaei4la8IaemOta40aaSbaaSqaaiabdogaJbqabaaabeaakiabgkHiTiabdweafjabcIcaOiab+feabjabcYcaSiab+neadjabcQcaQiabcMcaPiabc+caViabd6eaonaaBaaaleaacqWGJbWyaeqaaaGcbaGaeiiFaWNaemyrauKaeiikaGIae4xqaeKaeiilaWIae43qamKaeiOkaOIaeiykaKIaei4la8IaemOta40aaSbaaSqaaiabdogaJbqabaGccqGG8baFcqGGOaakcqaIXaqmcqGHsislcqWGXbqCdaWgaaWcbaGaeGimaadabeaakiabcMcaPaaacaWLjaGaaCzcamaabmaabaGaeGymaeJaeGynaudacaGLOaGaayzkaaaaaa@6EE6@

Here, *N*_c _is the number of contacts in the native structure, <*U*>_**A **_and *σ*_*U*,**A **_are the mean and standard deviation of all possible contact interactions in the protein sequence **A**, both native and non-native, *q*_0 _= 0.1 is a parameter representing the typical overlap of unrelated structures, *N *is chain length, *m*_*N *_is the number of alternative structures compatible with the above constraints, estimated through the empirical formula log(*m*_*N*_) ≈ 0.1 × *N *+ 4.

The above estimate may be improved considering that the probability of contact formation decreases with sequence distance [78]. However, this correction is small [47], and it will not be considered here because it would not allow us to get the analytic expression derived in the following section.

### Optimal hydrophobicity profile and the principal eigenvector of the contact matrix

We report here for completeness the calculation originally developed in [48] on the relationship between sequence and structural profiles induced by stability conditions.

The contact interaction matrix can be approximated through the main component of its spectral decomposition, *U*(*a, b*) ≈ - *h*(*a*)*h*(*b*). Here *h*(*a*) is the eigenvector of the matrix *U*(*a, b*) corresponding to the eigenvalue with the largest absolute value, which is negative. This eigenvector is very strongly correlated with the hydrophobicity of amino acid *a*, as for typical amino acid interaction matrices based on contacts [40,41]. We call the 20 parameters *h*(*a*) obtained from the principal eigenvector of the interaction matrix the *interactivity *hydrophobicity scale (IH), and we call the *N*-dimensional vector *h*(*A_i_*) the Hydrophobicity Profile (HP) of sequence **A **[48].

We use in the simulations the complete free energy function, Eq. (13), but in the analytic calculations we approximate it with the hydrophobic component,

H(A,C)≡−∑i<jCijh(Ai)h(Aj),     (16)
 MathType@MTEF@5@5@+=feaafiart1ev1aaatCvAUfKttLearuWrP9MDH5MBPbIqV92AaeXatLxBI9gBaebbnrfifHhDYfgasaacH8akY=wiFfYdH8Gipec8Eeeu0xXdbba9frFj0=OqFfea0dXdd9vqai=hGuQ8kuc9pgc9s8qqaq=dirpe0xb9q8qiLsFr0=vr0=vr0dc8meaabaqaciaacaGaaeqabaqabeGadaaakeaacqWGibascqGGOaakieqacqWFbbqqcqGGSaalcqWFdbWqcqGGPaqkcqGHHjIUcqGHsisldaaeqbqaaiabdoeadnaaBaaaleaacqWGPbqAcqWGQbGAaeqaaOGaemiAaGMaeiikaGIaemyqae0aaSbaaSqaaiabdMgaPbqabaGccqGGPaqkcqWGObaAcqGGOaakcqWGbbqqdaWgaaWcbaGaemOAaOgabeaakiabcMcaPaWcbaGaemyAaKMaeyipaWJaemOAaOgabeqdcqGHris5aOGaeiilaWIaaCzcaiaaxMaadaqadaqaaiabigdaXiabiAda2aGaayjkaiaawMcaaaaa@4FF6@

Using this approximation of the free energy, it is possible to derive an analytic relationship between the protein sequence and the protein structure. To this end, we calculate the optimally stable hydrophobicity profile, that minimizes the approximate effective free energy Eq. (16), for a given contact matrix, with a large normalized energy gap.

Using the REM estimate for the normalized energy gap, Eq. (15), we see that it depends on the protein sequence only through three parameters: the native energy and the mean and the standard deviation of the non-native contact interactions. Therefore, in order to minimize the native energy with a large normalized energy gap, we have to maintain fixed <*U*>_**A **_and *σ*_*U*,**A**_. Within the hydrophobic approximation of the contact interaction energy, it holds <*U*> ≈ <*h^2^*> and <*U*^2^>_**A **_≈ <*h*^2^>^2^.

In conclusion, we look for the hydrophobicity profile *h_i _*that minimizes the effective hydrophobic energy, Eq. (16), for a given contact matrix, and for given first and second moment of the hydrophobicity vector, <*h*> = *N*^-1 ^∑*_i_h*(*A_i_*) and <*h*^2^> = *N*^-1^∑_*i*_*h*(*A*_*i*_)^2^. In the calculation, we neglect the discretization in twenty values corresponding to natural amino acids.

The PE vi(1)
 MathType@MTEF@5@5@+=feaafiart1ev1aaatCvAUfKttLearuWrP9MDH5MBPbIqV92AaeXatLxBI9gBaebbnrfifHhDYfgasaacH8akY=wiFfYdH8Gipec8Eeeu0xXdbba9frFj0=OqFfea0dXdd9vqai=hGuQ8kuc9pgc9s8qqaq=dirpe0xb9q8qiLsFr0=vr0=vr0dc8meaabaqaciaacaGaaeqabaqabeGadaaakeaacqWG2bGDdaqhaaWcbaGaemyAaKgabaGaeiikaGIaeGymaeJaeiykaKcaaaaa@324B@ is the solution of the related minization problem in which no condition on <*h*> is imposed. We denote the eigenvalues of the contact matrix *C*_*ij *_by *λ*_*α *_and the corresponding eigenvectors by vi(α)
 MathType@MTEF@5@5@+=feaafiart1ev1aaatCvAUfKttLearuWrP9MDH5MBPbIqV92AaeXatLxBI9gBaebbnrfifHhDYfgasaacH8akY=wiFfYdH8Gipec8Eeeu0xXdbba9frFj0=OqFfea0dXdd9vqai=hGuQ8kuc9pgc9s8qqaq=dirpe0xb9q8qiLsFr0=vr0=vr0dc8meaabaqaciaacaGaaeqabaqabeGadaaakeaacqWG2bGDdaqhaaWcbaGaemyAaKgabaGaeiikaGccciGae8xSdeMaeiykaKcaaaaa@3301@. These eigenvectors constitute an orthonormal basis. Expressing the constraints on <*h*> and <*h*^2^> through Lagrange multipliers, one finds that the optimal HP is given by the following implicit expression,

hiopt=N〈h2〉∑αwαΛ−λαvi(α)∑αwα(Λ−λα)2     (17)
 MathType@MTEF@5@5@+=feaafiart1ev1aaatCvAUfKttLearuWrP9MDH5MBPbIqV92AaeXatLxBI9gBaebbnrfifHhDYfgasaacH8akY=wiFfYdH8Gipec8Eeeu0xXdbba9frFj0=OqFfea0dXdd9vqai=hGuQ8kuc9pgc9s8qqaq=dirpe0xb9q8qiLsFr0=vr0=vr0dc8meaabaqaciaacaGaaeqabaqabeGadaaakeaacqWGObaAdaqhaaWcbaGaemyAaKgabaGaee4Ba8MaeeiCaaNaeeiDaqhaaOGaeyypa0ZaaOaaaeaacqWGobGtcqGHPms4cqWGObaAdaahaaWcbeqaaiabikdaYaaakiabgQYiXdWcbeaakmaalaaabaWaaabeaeaadaWcaaqaamaakaaabaGaem4DaC3aaSbaaSqaaGGaciab=f7aHbqabaaabeaaaOqaaiabfU5amjabgkHiTiab=T7aSnaaBaaaleaacqWFXoqyaeqaaaaakiabdAha2naaDaaaleaacqWGPbqAaeaacqGGOaakcqWFXoqycqGGPaqkaaaabaGae8xSdegabeqdcqGHris5aaGcbaWaaOaaaeaadaaeqaqaamaalaaabaGaem4DaC3aaSbaaSqaaiab=f7aHbqabaaakeaacqGGOaakcqqHBoatcqGHsislcqWF7oaBdaWgaaWcbaGae8xSdegabeaakiabcMcaPmaaCaaaleqabaGaeGOmaidaaaaaaeaacqWFXoqyaeqaniabggHiLdaaleqaaaaakiaaxMaacaWLjaWaaeWaaeaacqaIXaqmcqaI3aWnaiaawIcacaGLPaaaaaa@63A9@

where the multiplier Λ is obtained through the constraint

τ≡〈h〉〈h2〉=∑αwαΛ−λα∑αwα(Λ−λα)2.     (18)
 MathType@MTEF@5@5@+=feaafiart1ev1aaatCvAUfKttLearuWrP9MDH5MBPbIqV92AaeXatLxBI9gBaebbnrfifHhDYfgasaacH8akY=wiFfYdH8Gipec8Eeeu0xXdbba9frFj0=OqFfea0dXdd9vqai=hGuQ8kuc9pgc9s8qqaq=dirpe0xb9q8qiLsFr0=vr0=vr0dc8meaabaqaciaacaGaaeqabaqabeGadaaakeaaiiGacqWFepaDcqGHHjIUdaWcaaqaaiabgMYiHlabdIgaOjabgQYiXdqaamaakaaabaGaeyykJeUaemiAaG2aaWbaaSqabeaacqaIYaGmaaGccqGHQms8aSqabaaaaOGaeyypa0ZaaSaaaeaadaaeqaqaamaalaaabaGaem4DaC3aaSbaaSqaaiab=f7aHbqabaaakeaacqqHBoatcqGHsislcqWF7oaBdaWgaaWcbaGae8xSdegabeaaaaaabaGae8xSdegabeqdcqGHris5aaGcbaWaaOaaaeaadaaeqaqaamaalaaabaGaem4DaC3aaSbaaSqaaiab=f7aHbqabaaakeaacqGGOaakcqqHBoatcqGHsislcqWF7oaBdaWgaaWcbaGae8xSdegabeaakiabcMcaPmaaCaaaleqabaGaeGOmaidaaaaaaeaacqWFXoqyaeqaniabggHiLdaaleqaaaaakiabc6caUiaaxMaacaWLjaWaaeWaaeaacqaIXaqmcqaI4aaoaiaawIcacaGLPaaaaaa@5E56@

*N *represents the number of residues in the protein and *w_α _*≡ N<*v*^(α)^>^2^= <*v*^(α)^>^2^/<(*v*^(α)^)^2^>, is the ratio between squared mean and mean square of the components of eigenvector *α*. Since the vi(α)
 MathType@MTEF@5@5@+=feaafiart1ev1aaatCvAUfKttLearuWrP9MDH5MBPbIqV92AaeXatLxBI9gBaebbnrfifHhDYfgasaacH8akY=wiFfYdH8Gipec8Eeeu0xXdbba9frFj0=OqFfea0dXdd9vqai=hGuQ8kuc9pgc9s8qqaq=dirpe0xb9q8qiLsFr0=vr0=vr0dc8meaabaqaciaacaGaaeqabaqabeGadaaakeaacqWG2bGDdaqhaaWcbaGaemyAaKgabaGaeiikaGccciGae8xSdeMaeiykaKcaaaaa@3301@ constitute an orthonormal basis, it holds ∑_*α *_*w_α _*= 1. The weight *w*_1 _of the principal eigenvector is the largest of the *w_α_*. The projection of the optimal HP along the direction of the PE is thus given by

∑ihioptvi(1)N〈h2〉=(1+(Λ−λ1)2w1∑α>1wα(Λ−λα)2)−1/2     (19)
 MathType@MTEF@5@5@+=feaafiart1ev1aaatCvAUfKttLearuWrP9MDH5MBPbIqV92AaeXatLxBI9gBaebbnrfifHhDYfgasaacH8akY=wiFfYdH8Gipec8Eeeu0xXdbba9frFj0=OqFfea0dXdd9vqai=hGuQ8kuc9pgc9s8qqaq=dirpe0xb9q8qiLsFr0=vr0=vr0dc8meaabaqaciaacaGaaeqabaqabeGadaaakeaadaWcaaqaamaaqababaGaemiAaG2aa0baaSqaaiabdMgaPbqaaiabb+gaVjabbchaWjabbsha0baakiabdAha2naaDaaaleaacqWGPbqAaeaacqGGOaakcqaIXaqmcqGGPaqkaaaabaGaemyAaKgabeqdcqGHris5aaGcbaWaaOaaaeaacqWGobGtcqGHPms4cqWGObaAdaahaaWcbeqaaiabikdaYaaakiabgQYiXdWcbeaaaaGccqGH9aqpdaqadaqaaiabigdaXiabgUcaRmaalaaabaGaeiikaGIaeu4MdWKaeyOeI0ccciGae83UdW2aaSbaaSqaaiabigdaXaqabaGccqGGPaqkdaahaaWcbeqaaiabikdaYaaaaOqaaiabdEha3naaBaaaleaacqaIXaqmaeqaaaaakmaaqafabaWaaSaaaeaacqWG3bWDdaWgaaWcbaGae8xSdegabeaaaOqaaiabcIcaOiabfU5amjabgkHiTiab=T7aSnaaBaaaleaacqWFXoqyaeqaaOGaeiykaKYaaWbaaSqabeaacqaIYaGmaaaaaaqaaiab=f7aHjabg6da+iabigdaXaqab0GaeyyeIuoaaOGaayjkaiaawMcaamaaCaaaleqabaGaeyOeI0IaeGymaeJaei4la8IaeGOmaidaaOGaaCzcaiaaxMaadaqadaqaaiabigdaXiabiMda5aGaayjkaiaawMcaaaaa@6D9F@

For Λ = *λ*_1_, the optimal HP is parallel to the PE, i.e. the coefficients of vi(α)
 MathType@MTEF@5@5@+=feaafiart1ev1aaatCvAUfKttLearuWrP9MDH5MBPbIqV92AaeXatLxBI9gBaebbnrfifHhDYfgasaacH8akY=wiFfYdH8Gipec8Eeeu0xXdbba9frFj0=OqFfea0dXdd9vqai=hGuQ8kuc9pgc9s8qqaq=dirpe0xb9q8qiLsFr0=vr0=vr0dc8meaabaqaciaacaGaaeqabaqabeGadaaakeaacqWG2bGDdaqhaaWcbaGaemyAaKgabaGaeiikaGccciGae8xSdeMaeiykaKcaaaaa@3301@ vanish for *α *> 1, as for the optimization without any constraint on <*h*>. In this case, *τ *= w1
 MathType@MTEF@5@5@+=feaafiart1ev1aaatCvAUfKttLearuWrP9MDH5MBPbIqV92AaeXatLxBI9gBaebbnrfifHhDYfgasaacH8akY=wiFfYdH8Gipec8Eeeu0xXdbba9frFj0=OqFfea0dXdd9vqai=hGuQ8kuc9pgc9s8qqaq=dirpe0xb9q8qiLsFr0=vr0=vr0dc8meaabaqaciaacaGaaeqabaqabeGadaaakeaadaGcaaqaaiabdEha3naaBaaaleaacqaIXaqmaeqaaaqabaaaaa@2F4F@, i.e. <*h*> = <*v*^(1)^> 〈h2〉/〈(v(1))2〉
 MathType@MTEF@5@5@+=feaafiart1ev1aaatCvAUfKttLearuWrP9MDH5MBPbIqV92AaeXatLxBI9gBaebbnrfifHhDYfgasaacH8akY=wiFfYdH8Gipec8Eeeu0xXdbba9frFj0=OqFfea0dXdd9vqai=hGuQ8kuc9pgc9s8qqaq=dirpe0xb9q8qiLsFr0=vr0=vr0dc8meaabaqaciaacaGaaeqabaqabeGadaaakeaadaGcaaqaaiabgMYiHlabdIgaOnaaCaaaleqabaGaeGOmaidaaOGaeyOkJeVaei4la8IaeyykJeUaeiikaGIaemODay3aaWbaaSqabeaacqGGOaakcqaIXaqmcqGGPaqkaaGccqGGPaqkdaahaaWcbeqaaiabikdaYaaakiabgQYiXdWcbeaaaaa@3E5E@, which means that the ratio between squared mean and mean square is the same for the HP and for the PE, and the energy is the same as in the absence of constraints on <*h*>, which is the lowest energy for all possible values of the constraint <*h*>.

A structure-derived quantity that estimates the importance of the minor eigenvectors with *α *> 1 in determining the optimal hydrophobicity profile is

η2=1w1∑α>1wα(λ1−λα)2,     (20)
 MathType@MTEF@5@5@+=feaafiart1ev1aaatCvAUfKttLearuWrP9MDH5MBPbIqV92AaeXatLxBI9gBaebbnrfifHhDYfgasaacH8akY=wiFfYdH8Gipec8Eeeu0xXdbba9frFj0=OqFfea0dXdd9vqai=hGuQ8kuc9pgc9s8qqaq=dirpe0xb9q8qiLsFr0=vr0=vr0dc8meaabaqaciaacaGaaeqabaqabeGadaaakeaaiiGacqWF3oaAdaWgaaWcbaGaeGOmaidabeaakiabg2da9maalaaabaGaeGymaedabaGaem4DaC3aaSbaaSqaaiabigdaXaqabaaaaOWaaabuaeaadaWcaaqaaiabdEha3naaBaaaleaacqWFXoqyaeqaaaGcbaWaaeWaaeaacqWF7oaBdaWgaaWcbaGaeGymaedabeaakiabgkHiTiab=T7aSnaaBaaaleaacqWFXoqyaeqaaaGccaGLOaGaayzkaaWaaWbaaSqabeaacqaIYaGmaaaaaaqaaiab=f7aHjabg6da+iabigdaXaqab0GaeyyeIuoakiabcYcaSiaaxMaacaWLjaWaaeWaaeaacqaIYaGmcqaIWaamaiaawIcacaGLPaaaaaa@4CAB@

In the SCN model, the values of <*h*> and <*h*^2^>, and therefore of *τ *and Λ, are not fixed by stability requirements but vary in a broad range depending on the mutation process and on the selection parameters. Numerical results show that, when *η*_2 _is small (smaller than, say, 0.1), the contribution of minor eigenvectors can be neglected in all the (quite broad) simulated range of parameters <*h*> and <*h*^2^>. This is the case of many single-domain proteins, since the *w_α _*corresponding to minor eigenvectors tend to be small in these cases. On the other hand, for modular (for instance multi-domains) structures, the *w_α _*of the eigenvectors that correspond to the minor domains are large, and these eigenvectors give a non-negligible contribution to the optimal HP.

For Λ ≈ *λ*_1 _and *τ *≈ w1
 MathType@MTEF@5@5@+=feaafiart1ev1aaatCvAUfKttLearuWrP9MDH5MBPbIqV92AaeXatLxBI9gBaebbnrfifHhDYfgasaacH8akY=wiFfYdH8Gipec8Eeeu0xXdbba9frFj0=OqFfea0dXdd9vqai=hGuQ8kuc9pgc9s8qqaq=dirpe0xb9q8qiLsFr0=vr0=vr0dc8meaabaqaciaacaGaaeqabaqabeGadaaakeaadaGcaaqaaiabdEha3naaBaaaleaacqaIXaqmaeqaaaqabaaaaa@2F4F@, it can be easily seen that the correlation coefficient between the optimal HP and the PE deviates from unity by a term of second order in 1−r(hiopt,vi(1))≈(τ/w1−1)2
 MathType@MTEF@5@5@+=feaafiart1ev1aaatCvAUfKttLearuWrP9MDH5MBPbIqV92AaeXatLxBI9gBaebbnrfifHhDYfgasaacH8akY=wiFfYdH8Gipec8Eeeu0xXdbba9frFj0=OqFfea0dXdd9vqai=hGuQ8kuc9pgc9s8qqaq=dirpe0xb9q8qiLsFr0=vr0=vr0dc8meaabaqaciaacaGaaeqabaqabeGadaaakeaacqaIXaqmcqGHsislcqWGYbGCcqGGOaakieqacqWFObaAdaqhaaWcbaGaemyAaKgabaGaee4Ba8MaeeiCaaNaeeiDaqhaaOGaeiilaWIae8NDay3aa0baaSqaaiabdMgaPbqaaiabcIcaOiabigdaXiabcMcaPaaakiabcMcaPiabgIKi7kabcIcaOGGaciab+r8a0jabc+caVmaakaaabaGaem4DaC3aaSbaaSqaaiabigdaXaqabaaabeaakiabgkHiTiabigdaXiabcMcaPmaaCaaaleqabaGaeGOmaidaaaaa@4B16@.

Even for extreme mutation bias, our simulation results yield *τ *w1
 MathType@MTEF@5@5@+=feaafiart1ev1aaatCvAUfKttLearuWrP9MDH5MBPbIqV92AaeXatLxBI9gBaebbnrfifHhDYfgasaacH8akY=wiFfYdH8Gipec8Eeeu0xXdbba9frFj0=OqFfea0dXdd9vqai=hGuQ8kuc9pgc9s8qqaq=dirpe0xb9q8qiLsFr0=vr0=vr0dc8meaabaqaciaacaGaaeqabaqabeGadaaakeaadaGcaaqaaiabdEha3naaBaaaleaacqaIXaqmaeqaaaqabaaaaa@2F4F@ ∈ [0.73, 0.97], which is close to one. Consistently, the constribution to minor eigenvectors is small in most of the range of parameters even for proteins for which *η*_2 _is large.

Therefore, we consider in this work the approximation that the correlation coefficient between the optimal HP and the PE is one, corresponding to the zeroth order in the *τ *- w1
 MathType@MTEF@5@5@+=feaafiart1ev1aaatCvAUfKttLearuWrP9MDH5MBPbIqV92AaeXatLxBI9gBaebbnrfifHhDYfgasaacH8akY=wiFfYdH8Gipec8Eeeu0xXdbba9frFj0=OqFfea0dXdd9vqai=hGuQ8kuc9pgc9s8qqaq=dirpe0xb9q8qiLsFr0=vr0=vr0dc8meaabaqaciaacaGaaeqabaqabeGadaaakeaadaGcaaqaaiabdEha3naaBaaaleaacqaIXaqmaeqaaaqabaaaaa@2F4F@ expansion or to a situation where *η*_2 _is very small. For simplicity of notation, the PE will be denoted in the following as *c*_*i *_≡ vi(1)
 MathType@MTEF@5@5@+=feaafiart1ev1aaatCvAUfKttLearuWrP9MDH5MBPbIqV92AaeXatLxBI9gBaebbnrfifHhDYfgasaacH8akY=wiFfYdH8Gipec8Eeeu0xXdbba9frFj0=OqFfea0dXdd9vqai=hGuQ8kuc9pgc9s8qqaq=dirpe0xb9q8qiLsFr0=vr0=vr0dc8meaabaqaciaacaGaaeqabaqabeGadaaakeaacqWG2bGDdaqhaaWcbaGaemyAaKgabaGaeiikaGIaeGymaeJaeiykaKcaaaaa@324B@. As a result, we get

hiopt≈〈h2〉−〈h〉2(〈c2〉−〈c〉2)(ci−〈c〉)+〈h〉.     (21)
 MathType@MTEF@5@5@+=feaafiart1ev1aaatCvAUfKttLearuWrP9MDH5MBPbIqV92AaeXatLxBI9gBaebbnrfifHhDYfgasaacH8akY=wiFfYdH8Gipec8Eeeu0xXdbba9frFj0=OqFfea0dXdd9vqai=hGuQ8kuc9pgc9s8qqaq=dirpe0xb9q8qiLsFr0=vr0=vr0dc8meaabaqaciaacaGaaeqabaqabeGadaaakeaacqWGObaAdaqhaaWcbaGaemyAaKgabaGaee4Ba8MaeeiCaaNaeeiDaqhaaOGaeyisIS7aaOaaaeaadaWcaaqaaiabgMYiHlabdIgaOnaaCaaaleqabaGaeGOmaidaaOGaeyOkJeVaeyOeI0IaeyykJeUaemiAaGMaeyOkJe=aaWbaaSqabeaacqaIYaGmaaaakeaadaqadaqaaiabgMYiHlabdogaJnaaCaaaleqabaGaeGOmaidaaOGaeyOkJeVaeyOeI0IaeyykJeUaem4yamMaeyOkJe=aaWbaaSqabeaacqaIYaGmaaaakiaawIcacaGLPaaaaaaaleqaaOGaeiikaGIaem4yam2aaSbaaSqaaiabdMgaPbqabaGccqGHsislcqGHPms4cqWGJbWycqGHQms8cqGGPaqkcqGHRaWkcqGHPms4cqWGObaAcqGHQms8cqGGUaGlcaWLjaGaaCzcamaabmaabaGaeGOmaiJaeGymaedacaGLOaGaayzkaaaaaa@66BD@

### Mutation process

The SCN model was originally defined at the protein sequence level, with equally probable mutations from one amino acid to any other one [30-32]. We have modified the mutation process in order to take into account the genetic code and the mutation bias at the DNA level (see also Ref. [53]). We represent each amino acid site by 3 nucleotides, and consider two mutation schemes: (1) Independent and identical mutation processes at each nucleotide site, each one satisfying detailed balance. (2) Same process as in (1), but with an enhanced mutation rate at CpG dinucleotides contained within a codon.

The mutation process (1) consists of the HKY mutation matrix [79,80] with rates Pμnuc
 MathType@MTEF@5@5@+=feaafiart1ev1aaatCvAUfKttLearuWrP9MDH5MBPbIqV92AaeXatLxBI9gBaebbnrfifHhDYfgasaacH8akY=wiFfYdH8Gipec8Eeeu0xXdbba9frFj0=OqFfea0dXdd9vqai=hGuQ8kuc9pgc9s8qqaq=dirpe0xb9q8qiLsFr0=vr0=vr0dc8meaabaqaciaacaGaaeqabaqabeGadaaakeaacqWGqbaudaqhaaWcbaacciGae8hVd0gabaacbaGae4NBa4Mae4xDauNae43yamgaaaaa@33E0@(*n*, *n'*) = *μf*(*n'*) if the mutation from *n *to *n' *is a transversion and Pμnuc
 MathType@MTEF@5@5@+=feaafiart1ev1aaatCvAUfKttLearuWrP9MDH5MBPbIqV92AaeXatLxBI9gBaebbnrfifHhDYfgasaacH8akY=wiFfYdH8Gipec8Eeeu0xXdbba9frFj0=OqFfea0dXdd9vqai=hGuQ8kuc9pgc9s8qqaq=dirpe0xb9q8qiLsFr0=vr0=vr0dc8meaabaqaciaacaGaaeqabaqabeGadaaakeaacqWGqbaudaqhaaWcbaacciGae8hVd0gabaacbaGae4NBa4Mae4xDauNae43yamgaaaaa@33E0@(*n*, *n'*) = *μkf*(*n'*) if it is a transition. Transitions are changes between the two purines A and G, or between the two pyrimidines C and T. Transversions are changes between a purine and a pyrimidine. Since they change less the chemical nature of the DNA basis, transitions are far more frequent than transversions. For convenience of notation, we define *t*(*n*) as the nucleotide obtained from *n *through a transition (*t*(A) = G, *t*(T) = C, *t*(*t*(*n*)) ≡ *n*). The diagonal elements of the mutation matrix are defined through the normalization condition Pμnuc
 MathType@MTEF@5@5@+=feaafiart1ev1aaatCvAUfKttLearuWrP9MDH5MBPbIqV92AaeXatLxBI9gBaebbnrfifHhDYfgasaacH8akY=wiFfYdH8Gipec8Eeeu0xXdbba9frFj0=OqFfea0dXdd9vqai=hGuQ8kuc9pgc9s8qqaq=dirpe0xb9q8qiLsFr0=vr0=vr0dc8meaabaqaciaacaGaaeqabaqabeGadaaakeaacqWGqbaudaqhaaWcbaacciGae8hVd0gabaacbaGae4NBa4Mae4xDauNae43yamgaaaaa@33E0@(*n*, *n*) = 1 - ∑_*n' *≠ *n *_Pμnuc
 MathType@MTEF@5@5@+=feaafiart1ev1aaatCvAUfKttLearuWrP9MDH5MBPbIqV92AaeXatLxBI9gBaebbnrfifHhDYfgasaacH8akY=wiFfYdH8Gipec8Eeeu0xXdbba9frFj0=OqFfea0dXdd9vqai=hGuQ8kuc9pgc9s8qqaq=dirpe0xb9q8qiLsFr0=vr0=vr0dc8meaabaqaciaacaGaaeqabaqabeGadaaakeaacqWGqbaudaqhaaWcbaacciGae8hVd0gabaacbaGae4NBa4Mae4xDauNae43yamgaaaaa@33E0@(*n, n'*). This mutation process satisfies detailed balance, with stationary distribution given by the frequencies *f*(*n*) independently of the transition-tranversion ratio *k *that, therefore, is expected to have no influence on the stationary amino acid distribution as well.

In SCN simulations, in order to reduce the number of parameters, we further imposed the condition that the mutation process is the same on the two DNA strands, so that *f*(G) ≡ *f*(C) and *f*(A) ≡ *f*(T). Therefore, the stationary distributions only depend on the GC bias *f*(G)/*f*(A).

The mutation process (2) starts from the same model as in (1), but every time a codon contains a CpG dinucleotide the rate of the mutations from C to T and from G to A are enhanced by a factor *k*_CpG _≥ 1. This model was considered with a transition-transversion ratio *k *= 1, and it was only used in calculations with the mean-field model, and not in SCN simulations. For simplicity, only CpG dinucleotides within a codon were considered, so that we obtained an independent mutation process for each codon. From the above definition, we computed the mutation matrix at the codon level to be used in the master equation (8), PμCOD
 MathType@MTEF@5@5@+=feaafiart1ev1aaatCvAUfKttLearuWrP9MDH5MBPbIqV92AaeXatLxBI9gBaebbnrfifHhDYfgasaacH8akY=wiFfYdH8Gipec8Eeeu0xXdbba9frFj0=OqFfea0dXdd9vqai=hGuQ8kuc9pgc9s8qqaq=dirpe0xb9q8qiLsFr0=vr0=vr0dc8meaabaqaciaacaGaaeqabaqabeGadaaakeaacqWGqbaudaqhaaWcbaacciGae8hVd0gabaacbaGae43qamKae43ta8Kae4hraqeaaaaa@3300@(*n*_1_*n*_2_*n*_3_,n′1n′2n′3
 MathType@MTEF@5@5@+=feaafiart1ev1aaatCvAUfKttLearuWrP9MDH5MBPbIqV92AaeXatLxBI9gBaebbnrfifHhDYfgasaacH8akY=wiFfYdH8Gipec8Eeeu0xXdbba9frFj0=OqFfea0dXdd9vqai=hGuQ8kuc9pgc9s8qqaq=dirpe0xb9q8qiLsFr0=vr0=vr0dc8meaabaqaciaacaGaaeqabaqabeGadaaakeaacuWGUbGBgaqbamaaBaaaleaacqaIXaqmaeqaaOGafmOBa4MbauaadaWgaaWcbaGaeGOmaidabeaakiqbd6gaUzaafaWaaSbaaSqaaiabiodaZaqabaaaaa@346D@). The matrix element is set to zero if the two codons differ at more that one position and to Pμnuc
 MathType@MTEF@5@5@+=feaafiart1ev1aaatCvAUfKttLearuWrP9MDH5MBPbIqV92AaeXatLxBI9gBaebbnrfifHhDYfgasaacH8akY=wiFfYdH8Gipec8Eeeu0xXdbba9frFj0=OqFfea0dXdd9vqai=hGuQ8kuc9pgc9s8qqaq=dirpe0xb9q8qiLsFr0=vr0=vr0dc8meaabaqaciaacaGaaeqabaqabeGadaaakeaacqWGqbaudaqhaaWcbaacciGae8hVd0gabaacbaGae4NBa4Mae4xDauNae43yamgaaaaa@33E0@(*n*, *n'*) if the two codons differ at one position where they contain respectively nucleotides *n *and *n'*. If the mutated nucleotide is either the C or the G of a CpG dinucleotide contained into codon *n*_1_*n*_2_*n*_3 _and the mutation is a transition (C to T or G to A), then the matrix element is increased by a factor *k*_CpG._

In the SCN model, the mutation process is simulated extracting at random at each time step the site where a mutation takes place. The probability that a site is extracted depends on the nucleotide occupying it, and it is *p*(*n*) = ∑_*n' *≠ *n *_*f*(*n'*) = (*k *- 1)*f*(*t*(*n*)).

### Calculation of the mean-field distributions

The mean-field amino acid distributions were computed in two steps. In a first step, we computed the site-specific mean hydrophobicities [*h*_*i*_] using Eq. (5), which needs as input the distribution of PE values, i.e. the fraction of sites with *c*_*i*_/<*c*> in a given range, and the mean and standard deviation of the hydrophobicity, which were obtained from the protein sequences. In a second step, and for a given mutation model, the site-specific mean-field distributions were calculated as a function of *β *and a value *β_i _*was associated to each site in such a way that the mean hydrophobicity at the site coincides with the predicted one (numerically, the predicted value of [*h_i_*] was bounded between an upper and a lower bound corresponding to two *β *values, and *β_i _*was found by interpolation).

The mean hydrophobicity was calculated as [*h*]*β *= ∑_*a*_*h*(*a*)*π*(*β*, *a*). For mutation models fulfilling detailed balance, we used Eq. (10), *π*(*β*, *a*) ∝ *w*_AA _exp [-(*β h*(*a*)], with weights *w*_AA_(*a*) obtained from the mutation model. For mutation models not obeying detailed balance, *π*(*β*, *a*) was numerically computed as the stationary distribution of the Markov process, Eq. (8).

### Computation of the acceptance rate

The rate of acceptance of a mutation at position *i *in the stationary state was calculated as

Pacc,i=∑nn′PiCOD(n)PμCOD(n,n′)min⁡(1,exp⁡[−βi[h(A[n′])−h(A[n])]])∑nn′PiCOD(n)PμCOD(n,n′),     (22)
 MathType@MTEF@5@5@+=feaafiart1ev1aaatCvAUfKttLearuWrP9MDH5MBPbIqV92AaeXatLxBI9gBamrtHrhAL1wy0L2yHvtyaeHbnfgDOvwBHrxAJfwnaebbnrfifHhDYfgasaacH8akY=wiFfYdH8Gipec8Eeeu0xXdbba9frFj0=OqFfea0dXdd9vqai=hGuQ8kuc9pgc9s8qqaq=dirpe0xb9q8qiLsFr0=vr0=vr0dc8meaabaqaciaacaGaaeqabaWaaeGaeaaakeaacqWGqbaudaWgaaWcbaGaeeyyaeMaee4yamMaee4yamMaeiilaWIaemyAaKgabeaakiabg2da9maalaaabaWaaabeaeaacqWGqbaudaqhaaWcbaGaemyAaKgabaGaee4qamKaee4ta8KaeeiraqeaaaqaaGqabiab=5gaUjqb=5gaUzaafaaabeqdcqGHris5aOGaeiikaGIae8NBa4MaeiykaKIaemiuaa1aa0baaSqaaGGaciab+X7aTbqaaiabboeadjabb+eapjabbseaebaakiabcIcaOiab=5gaUjabcYcaSiqb=5gaUzaafaGaeiykaKIagiyBa0MaeiyAaKMaeiOBa4MaeiikaGIaeGymaeJaeiilaWIagiyzauMaeiiEaGNaeiiCaaNaei4waSLaeyOeI0Iae4NSdi2aaSbaaSqaaiabdMgaPbqabaGccqGGBbWwcqWGObaAcqGGOaakimaacqqFaeFqcqGGBbWwcuWFUbGBgaqbaiabc2faDjabcMcaPiabgkHiTiabdIgaOjabcIcaOiab9bq8bjabcUfaBjab=5gaUjabc2faDjabcMcaPiabc2faDjabc2faDjabcMcaPaqaamaaqababaGaemiuaa1aa0baaSqaaiabdMgaPbqaaiabboeadjabb+eapjabbseaebaaaeaacqWFUbGBcuWFUbGBgaqbaaqab0GaeyyeIuoakiabcIcaOiab=5gaUjabcMcaPiabdcfaqnaaDaaaleaacqGF8oqBaeaacqqGdbWqcqqGpbWtcqqGebaraaGccqGGOaakcqWFUbGBcqGGSaalcuWFUbGBgaqbaiabcMcaPaaacqGGSaalcaWLjaGaaCzcamaabmaabaGaeGOmaiJaeGOmaidacaGLOaGaayzkaaaaaa@9E9C@

where we use the notation introduced previously, PiCOD
 MathType@MTEF@5@5@+=feaafiart1ev1aaatCvAUfKttLearuWrP9MDH5MBPbIqV92AaeXatLxBI9gBaebbnrfifHhDYfgasaacH8akY=wiFfYdH8Gipec8Eeeu0xXdbba9frFj0=OqFfea0dXdd9vqai=hGuQ8kuc9pgc9s8qqaq=dirpe0xb9q8qiLsFr0=vr0=vr0dc8meaabaqaciaacaGaaeqabaqabeGadaaakeaacqWGqbaudaqhaaWcbaGaemyAaKgabaGaee4qamKaee4ta8Kaeeiraqeaaaaa@329E@ (**n**) is the stationary frequency of codon **n **in the mean-field model, and the summations exclude stop codons.

### Observed amino acid distributions

We compared our predictions to site-specific distributions sampled from a representative subset of the Protein Data Bank (PDB). We considered a non-redundant subset of single-domain globular proteins in the PDB, with a sequence identity below 25% [58]. Globularity was verified by imposing that the fraction of contacts per residue was larger than a length dependent threshold, *N*_*c*_/*N *> 3.5 + 7.8*N*^-1/3^. This functional form represents the scaling of the number of contacts in globular proteins as a function of chain length (the factor *N*^-1/3 ^comes from the surface to volume ratio), and the two parameters were chosen so as to eliminate outliers with respect to the general trend, which are mainly non-globular structures. The condition of being single-domain was verified by imposing that the normalized variance of the PE components was smaller than a threshold, (1 - *N*<*c*>^2^)/(*N*<*c*>^2^) < 1.5. Multi-domain proteins have PE components which are large inside the main domain and small outside it. The PE components would be exactly zero outside the main domain if the domains do not share contacts (see for instance Ref. [61]). Therefore, multi-domain proteins are characterized by a larger normalized variance of PE components with respect to single-domain ones. We have verified that the threshold of 1.5 is able to eliminate most of the known multi-domain proteins and very few of the known single-domain proteins (data not shown). We selected 404 such structures with 200 or less amino acids. We counted the number of each of the 20 amino acids as a function of *c*_*i*_/<*c*>, where <*c*> denotes the average over a single structure. We used a bin-size of 0.05 for *c*_*i*_/<*c*> ≤ 2.5 and a bin-size of 0.1 for *c*_*i*_/<*c*> > 2.5.

### Similarity score between observed and predicted amino acid distributions

The accuracy of the predicted amino acid distributions was assessed by calculating the mean correlation coefficient between observed and predicted amino acid distributions for *M *structures, given by

〈r〉=M−1∑i=1Mr(πci/〈c〉obs,πci/〈c〉pred)
 MathType@MTEF@5@5@+=feaafiart1ev1aaatCvAUfKttLearuWrP9MDH5MBPbIqV92AaeXatLxBI9gBaebbnrfifHhDYfgasaacH8akY=wiFfYdH8Gipec8Eeeu0xXdbba9frFj0=OqFfea0dXdd9vqai=hGuQ8kuc9pgc9s8qqaq=dirpe0xb9q8qiLsFr0=vr0=vr0dc8meaabaqaciaacaGaaeqabaqabeGadaaakeaacqGHPms4cqWGYbGCcqGHQms8cqGH9aqpcqWGnbqtdaahaaWcbeqaaiabgkHiTiabigdaXaaakmaaqadabaGaemOCai3aaeWaaeaaiiGacqWFapaCdaqhaaWcbaGaem4yam2aaSbaaWqaaiabdMgaPbqabaWccqGGVaWlcqGHPms4cqWGJbWycqGHQms8aeaacqqGVbWBcqqGIbGycqqGZbWCaaGccqGGSaalcqWFapaCdaqhaaWcbaGaem4yam2aaSbaaWqaaiabdMgaPbqabaWccqGGVaWlcqGHPms4cqWGJbWycqGHQms8aeaacqqGWbaCcqqGYbGCcqqGLbqzcqqGKbazaaaakiaawIcacaGLPaaaaSqaaiabdMgaPjabg2da9iabigdaXaqaaiabd2eanbqdcqGHris5aaaa@5ED2@

### Optimization of the mutation parameters

The optimal values for the parameters of the different mutation models were found by maximizing the mean correlation coefficient <*r*> between observed and predicted amino acid distributions as defined in the previous subsection. First, we discretized the possible values of the free parameters within a reasonable range, using a step size of 0.001, and we numerically assessed all possible combinations. We then performed a (much faster) optimization by gradient descent, finding the same results up to relative precision of 10^-3^.

### Hydropathy scales

In this work, mean-field distributions were obtained using interactivity (IH, see below) as hydrophobicity scale *h*(a), both for comparison with SCN simulations and with amino acid distributions sampled from the PDB. However, for the latter case we tested eleven hydropathy scales, finding that all other scales provide worse results. They are: (1) The KD82 hydropathy scale, derived to identify trans-membrane helices using diverse experimental data [81]; (2) The L76 hydropathy scale, which was derived by using experimental data and theoretical calculations [82]; (3) The R88 hydropathy scale, which is based on the transfer of solutes from water to alkane solvents [83]; (4) The augmented Whilmey-White (WW01) hydropathy scale, derived to improve recognition of trans-membrane helices [84]; (5) The G98 classification of amino acids into polar, hydrophobic, and amphiphylic classes, adopted by Gu et al. [85] to investigate the relationship between the hydrophobicity of a protein and the nucleotide composition of the corresponding gene; (6) The MP78 hydropathy scale, derived from statistical properties of globular proteins [86]; (7) The AV hydropathy scale, derived by averaging 127 normalized hydropathy scales published in the literature [87]; (8) The FP83 hydropathy scale, derived from the experimental measurement of octanol/water partition coefficients [42]; (9) The ZZ04 scale, also called buriability, proposed by Zhou and Zhou [59]; (10) The interaction scale IH, obtained from the main eigenvector of the interaction matrix *U*(*a, b*) used in this work [48]; (11) The optimized interactivity scale, or connectivity scale CH, which maximizes the correlation with the principal eigenvector of protein contact matrices for a non-redundant set of Protein Data Bank (PDB) structures [48].

## Authors' contributions

UB wrote the code for the SCN simulations, developed the mean-field model, analyzed PDB sequences with the mean-field model, contributed to the analysis of the data and wrote the first version of the paper. MP performed the SCN simulations, analyzed PDB sequences with the mean-field model, and contributed to the analysis of the data and the writing of the paper. HER contributed to the analysis of the data and the writing of the paper. MV contributed to the analysis of the data and the writing of the paper.

## Note

^1 ^Here and in the following, the angular brackets (·) denote the average over all sites in the protein.

^2^We also verified that, in agreement with Eq. (17), when other eigenvectors are relevant their scalar product with the average HP is correlated with the factor *w_α_*/(*λ*_1 _- *λ*_*α*_). For myoglobin the correlation coefficients, for the 15 most relevant eigenvectors excluding the PE, are in the range between 0.73 and 0.93, except for the extreme mutation bias, for ATPE they are always larger than 0.81.
